# Occurrence of a d-arabinose-containing complex-type free-*N*-glycan in the urine of cancer patients

**DOI:** 10.1038/s41598-022-08790-0

**Published:** 2022-03-22

**Authors:** Miki Tanaka-Okamoto, Ken Hanzawa, Hiroko Murakami, Mikio Mukai, Hidenori Takahashi, Takeshi Omori, Kenji Ikezawa, Kazuyoshi Ohkawa, Masayuki Ohue, Yasuhide Miyamoto

**Affiliations:** 1 Department of Molecular Biology, Osaka International Cancer Institute, 3-1-69 Otemae, Chuo-ku, Osaka, 541-8567 Japan; 2 Department of Medical Checkup, Osaka International Cancer Institute, 3-1-69 Otemae, Chuo-ku, Osaka, 541-8567 Japan; 3 Department of Gastroenterological Surgery, Osaka International Cancer Institute, 3-1-69 Otemae, Chuo-ku, Osaka, 541-8567 Japan; 4 Department of Hepatobiliary and Pancreatic Oncology, Osaka International Cancer Institute, 3-1-69 Otemae, Chuo-ku, Osaka, 541-8567 Japan

**Keywords:** Biochemistry, Cancer, Biomarkers, Oncology

## Abstract

Urinary free-glycans are promising markers of disease. In this study, we attempted to identify novel tumor markers by focusing on neutral free-glycans in urine. Free-glycans extracted from the urine of normal subjects and cancer patients with gastric, colorectal, pancreatic and bile duct were fluorescently labeled with 2-aminopyridine. Profiles of these neutral free-glycans constructed using multidimensional high performance liquid chromatography separation were compared between normal controls and cancer patients. The analysis identified one glycan in the urine of cancer patients with a unique structure, which included a pentose residue. To reveal the glycan structure, the linkage fashion, monosaccharide species and enantiomer of the pentose were analyzed by high performance liquid chromatography and mass spectrometry combined with several chemical treatments. The backbone of the glycan was a monoantennary complex-type free-*N*-glycan containing β1,4-branch. The pentose residue was attached to the antennal GlcNAc and released by α1,3/4-l-fucosidase. Intriguingly, the pentose residue was consistent with d-arabinose. Collectively, this glycan structure was determined to be Galβ1-4(d-Araβ1-3)GlcNAcβ1-4Manα1-3Manβ1-4GlcNAc-PA. Elevation of d-arabinose-containing free-glycans in the urine of cancer patients was confirmed by selected reaction monitoring. This is the first study to unequivocally show the occurrence of a d-arabinose-containing oligosaccharide in human together with its detailed structure.

## Introduction

Glycans in living organisms, which exist on the cell surface in the form of complex carbohydrates attached to proteins and lipids, act as modulators of intercellular communication^1^. These glycans also play a role in the cellular protein quality-control system^2,3^. During the protein quality-control process in the endoplasmic reticulum, misfolded proteins are discharged into the cytoplasm. Misfolded protein-attached glycans are thought to be detached by enzymatic digestion and transported from the cytoplasm to lysosomes, where they are degraded into monosaccharides by lysosomal enzymes. However, it has been reported that some oligosaccharides exist as free forms in the intracellular and extracellular regions without being bound to proteins or lipids^4–6^. Indeed, a large amount of free-glycan is detected in urine of patients with lysosomal storage disease caused by deficiency of lysosomal enzymes, suggesting that urinary free-glycans may be disease markers^7–9^.

It is known that glycan structures on the cell surface are altered during malignant transformation. Therefore, glycan structures such as sialyl Lewis A and sialyl Tn, which are present in cancer cells, have been used as tumor markers for clinical diagnosis. To identify novel glycan tumor markers, we previously conducted *O*-glycan profiling of human sera by multi-step high performance liquid chromatography (HPLC) analysis^10^, which detected a group of acidic *O*-glycan tumor marker candidates that are predominantly elevated in the sera of cancer patients^11–13^. Moreover, earlier glycomics analysis of cancer tissues had revealed an unusual accumulation of *N*-acetylneuraminic acid (Neu5Ac)- and deaminoneuraminic acid (KDN)-linked *N*-type free-glycans^14,15^. Taken together, these results suggested that free-glycans might be candidate tumor markers. Importantly, free-glycans are rapidly excreted from the bloodstream into the urine. Hence, in an effort to identify novel tumor markers we have conducted precise structural analyses of free-glycans found in urine. Our initial studies focused on identifying tumor markers comprising acidic free-glycans. The structures of 135 free-glycans were identified, and several free-glycans that were elevated in the urine of gastric, pancreatic cancer and cholangiocarcinoma patients found^16^. In a subsequent study, structural analyses of neutral free-glycan in urine of healthy subjects were conducted^17^. This analysis revealed the presence of novel glycans, β1,3-galactosylglucose-core (Galβ1-3Glc) glycans, as major components, in addition to previously reported neutral free-glycans such as lactose core (Galβ1-4Glc) glycans, type II *N*-acetyllactosamine core (Galβ1-4GlcNAc) glycans, hexose oligomers, *N*-glycans.

Here, the profiles of neutral free-glycans in normal subjects and patients with gastric, colorectal, pancreatic and bile duct cancer were compared in an attempt to identify neutral tumor marker candidates. The analysis identified a novel pentose-containing complex-type free-*N*-glycan that was present at elevated levels in the urine of cancer patients. The linkage fashion, monosaccharide species and enantiomer of the pentose residue were determined using various approaches such as periodate oxidation, permethylation, derivatization of the isolated pentose and α1,3/4-l-fucosidase treatment. The backbone structure of the glycan was analyzed using acid hydrolysis, and enzymatic digestion. The results of the analysis showed the pentose to be d-arabinose that was β1,3-linked to the subterminal GlcNAc of the Galβ1-4GlcNAcβ1-4Manα1-3Manβ1-4GlcNAc backbone. Intriguingly, d-arabinose has not been reported to be biosynthesized in humans. Indeed, to date, there have been few studies showing the unequivocal occurrence of d-arabinose-containing glycan in human or its precise structure. Finally, the quantification of this d-arabinose-containing glycan by selected reaction monitoring (SRM) confirmed that it was elevated in cancer patients.

## Results

### Preparation and separation of PA-labeled neutral free-glycans derived from urine

**Figure 1:**
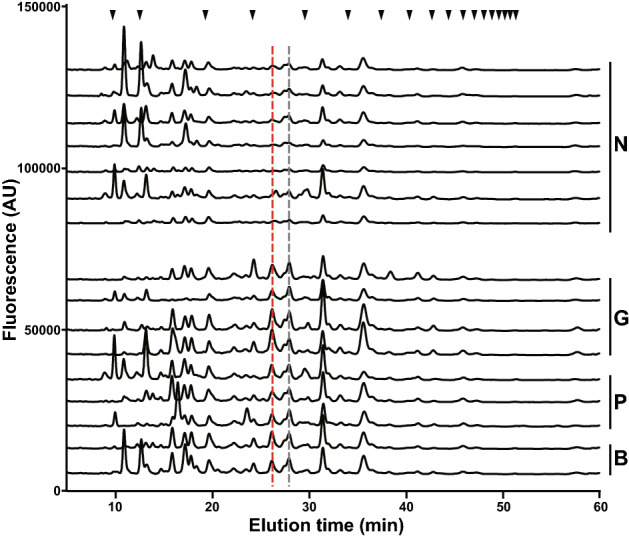
Reversed phase-HPLC chromatograms of PA-labeled neutral free-glycans prepared from the urine of normal individuals and cancer patients. PA-labeled neutral free-glycans fractionated by NP-HPLC were further separated by RP-HPLC. Chromatograms corresponding to RP-HPLC of NP-fraction 8 eluted by NP-HPLC are shown. These chromatograms contain neutral free-glycans in urine equivalent to 50 μg of creatinine. Representative chromatograms of seven normal individuals (N6, N7, N11, N14, N15, N19, N21), four gastric cancer patients (G2, G5, G8, G10), three pancreatic cancer patients (P2, P3, P5) and two bile duct cancer patients (B2, B4) are shown. The red dotted line indicates the peak containing the pentose-attached glycan, which was increased in cancer patients. The gray dotted line peak contains the xylose- and fucose-attached glycans measured by SRM (see Fig. 6). The black inverted triangles represent glucose units based on the elution time of PA-labeled isomaltooligosaccharides.

To search for potential tumor markers in human urine, urine specimens were obtained from 22 gastric, 25 colorectal, 11 pancreatic and 4 bile duct cancer patients and 21 normal subjects, which were collected in the early morning after fasting. Clinical information on all specimens is shown in Supplementary Table S1. Urinary free-glycans were prepared as previously reported for acidic glycans^16^. The reducing ends of the free-glycans purified from creatinine-corrected urine were labeled with a fluorophore, 2-aminopyridine (PA), by a reductive amination reaction. The PA-glycan mixture was separated according to charge by DEAE anion-exchange HPLC, and the neutral fractions collected. The neutral glycan mixture was fractionated into twelve fractions by normal phase (NP)-HPLC (NP-fractions 1–12, Supplementary Fig. S1), and each NP-fraction was further separated by reversed phase (RP)-HPLC. The elution patterns of each chromatogram of RP-HPLC obtained from cancer patient specimens was exhaustively compared with those of normal subjects (Fig. 1). Peaks markedly elevated in the cancer specimens were identified as potential tumor marker candidates and subjected to MS/MS.

### Identification of the pentose-containing free-N-glycan elevated in the urine of cancer patients

**Figure 2:**
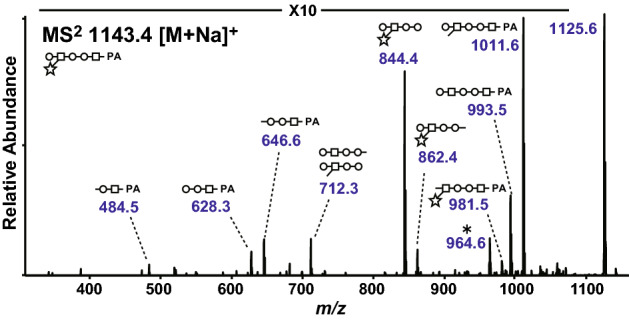
LC–MS/MS of the pentose-containing glycan. MS^2^ spectrum of the pentose-containing PA-glycan at *m/z* 1143 [M + Na]^+^ is shown. Symbol representations of glycans are as follows: circle, hexose; square, HexNAc; star, pentose. The asterisk indicates an unassigned fragment mass.

Most urinary neutral free-glycans corresponded to ABO-blood group antigens as previously reported in our analysis of normal urine specimens^17^. Comparison of free-glycan profiles in the urine from normal subjects and cancer patients identified several peaks that were elevated in cancer patients. As expected, most of these elevated peaks were composed of glycan structures with blood group antigens. However, free-glycans with ABO-antigens were unreliable as tumor marker candidates because there was significant variability in the level of these glycans across only a small number of patients. Therefore, we focused on blood type-independent glycans as potential tumor marker candidates. A peak that was clearly elevated in the urine of several cancer patients, but present at negligible levels in normal subjects, was observed in RP-chromatograms of NP-fraction 8 (Fig. 1, red dotted line). This peak was primarily composed of a single glycan species with a unique structure. The spectrum obtained from LC–MS/MS showed that this novel glycan structure was composed of Hex_3_HexNAc_2_Pen_1_-PA (*m/z* 1143 [M + Na]^+^), comprising a Hex-HexNAc-Hex-Hex-HexNAc backbone with a pentose residue and pentose-containing free-Gn1 *N*-glycan (Fig. 2).

### Structural analyses of the pentose-containing free-N-glycan

**Figure 3:**
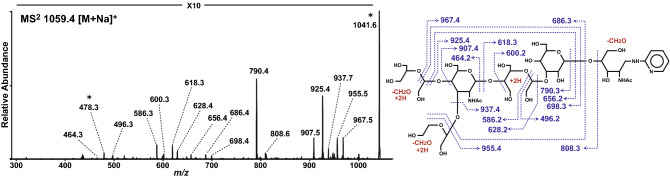
LC–MS/MS of the pentose-containing glycan after periodate cleavage. The periodate oxidation products were analyzed by LC–MS/MS. MS^2^ spectrum of the periodate-cleaved hexasaccharide at *m/z* 1059 [M + Na]^+^ (Hex_3_HexNAc_2_Pen_1_-PA + 3 × 2H, − 3 × CH_2_O) in positive ion mode MS is shown. The MS^2^ fragment ions were assigned as shown in the illustration. Assignments were based on a combination of results from other structural analyses. Note, it was not possible to determine whether the linkage positions of the terminal hexose and pentose residues was C-3 or C-4, respectively. Asterisks indicate dehydrated products.

**Figure 4:**
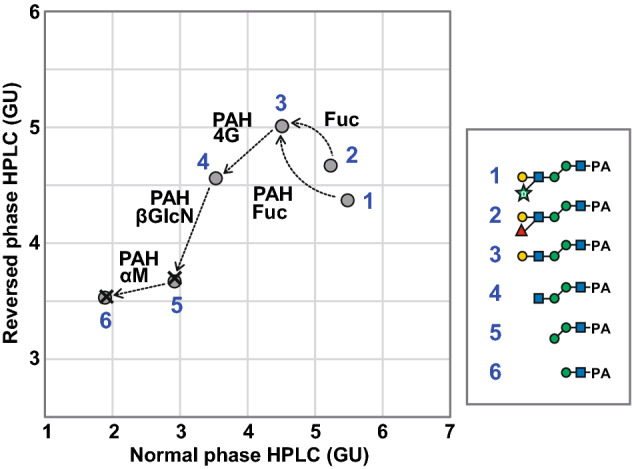
Two-dimensional mapping of the pentose-containing *N*-glycan treated by partial acid hydrolysis or sequential enzymatic digestions. Elution positions of PA-glycans on normal phase- and reversed phase-HPLC are represented by glucose units (GU). Closed gray circles indicate the position of the pentose-containing PA-glycan or its products by partial acid hydrolysis (PAH) or sequential enzymatic digestions. No. 2 used as a standard was an enzymatic digestion product of triantennary *N*-glycan obtained from α1-acid glycoprotein. × marks the positions of the authentic standard compounds. Enzymes used for glycan structural analyses are abbreviated as follows: Fuc, α1,3/4-l-fucosidase; 4G, β4-d-Galactosidase; βGlcN, *N*-acetyl-β-d-glucosaminidase S; αM, α1,2/3/6-mannosidase. Symbol representations of glycans are as follows; galactose, yellow circle; mannose, green circle; GlcNAc, blue square; arabinose, green star; fucose, red triangle.

The structure of this characteristic glycan was determined using periodate oxidation, partial acid hydrolysis (PAH), enzymatic digestion and permethylation. Periodate oxidation products were subjected to MS with sodium adduct ions for inhibition of pentose migration^18,19^. The MS^2^ spectrum of the periodate-cleaved product from the sodiated precursor ion at *m/z* 1059 indicated the glycan structure was Hex1-3/4(Pen1-3/4)HexNAc1-4Hex1-3Hex1-4HexNAc-PA (Fig. 3). The pentose residue was subsequently released by PAH with 0.1 M TFA. The backbone structure with the pentose removed corresponded to the authentic standard, Galβ1-4GlcNAcβ1-4Manα1-3Manβ1-4GlcNAc-PA (Fig. 4 and Supplementary Fig. S2), but not the other Gn1 free-glycans, Galβ1-4GlcNAcβ1-2Manα1-3Manβ1-4GlcNAc-PA and Galβ1-4GlcNAcβ1-2Manα1-6Manβ1-4GlcNAc-PA. The backbone structure was also confirmed by sequential enzymatic digestions of the PAH product, Hex_3_HexNAc_2_-PA, with β4-galactosidase, *N*-acetyl-β-glucosaminidase and α-mannosidase (Fig. 4). Additionally, permethylation of this glycan was performed to determine the linkage position of the pentose residue. Permethylated products were subjected to MS with sodium adduct ions and detected as a precursor ion at *m/z* 1423. The MS^3^ spectrum of the antennal B-ion precursor at *m/z* 646 corresponding to the terminal Hex_1_HexNAc_1_Pen_1_ from MS^2^ spectrum showed 2,4- and 3,5-cross-ring cleavage ions at *m/z* 301 and 329, respectively (Supplementary Fig. S3). This analysis suggested substitutions of the C-3 and C-4 positions of HexNAc, and that the hexose residue must be linked to the C-4 position of HexNAc. Specifically, the pentose residue must be linked to the C-3 position of the subterminal GlcNAc residue. Taken together, the results suggested that this oligosaccharide corresponded to Galβ1-4(Pen1-3)GlcNAcβ1-4Manα1-3Manβ1-4GlcNAc-PA, Gn1 type *N*-glycan.

### Identification of the pentose monosaccharide linked to the free-N-glycans

**Figure 5:**
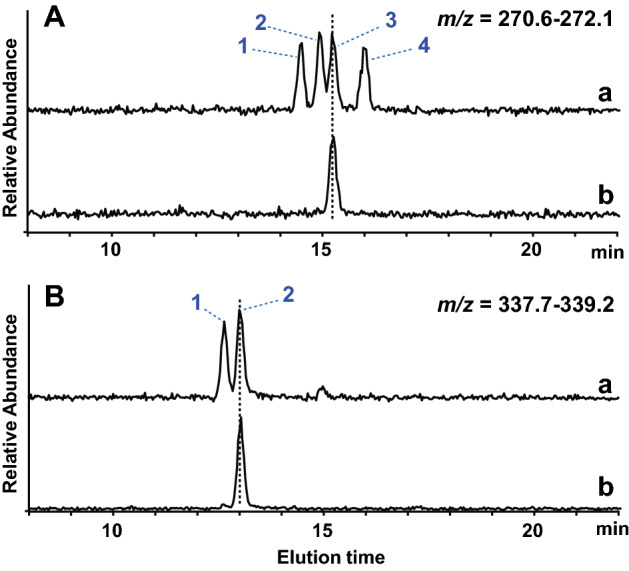
LC–MS analysis of the pentose monosaccharide released from the pentose-containing glycan. The pentose residue liberated from the pentose-containing *N*-glycan was labeled with 2AB or l-TrpNH_2_ and then subjected to LC–MS. (**A**) Extracted ion chromatography (mass ranges *m/z* 270.6–272.1) of 2AB-labeled pentoses. (a) Chromatograph of the pentose monosaccharide mixture used as a standard. The blue numbers denote 2AB-ribose (1), 2AB-lyxose (2), 2AB-arabinose (3) and 2AB-xylose (4). The chromatogram of the pentose monosaccharide released from the pentose-containing *N*-glycan by partial acid hydrolysis is in (b). (**B**) Extracted ion chromatography (mass ranges *m/z* 337.7–339.2) of l-TrpNH_2_-arabinose. l-TrpNH_2_-derivatized l-arabinose (1) and d-arabinose (2) were utilized as standards (a). (b) Chromatograph of an l-TrpNH_2_-derivative of arabinose released from the original *N*-glycan by partial acid hydrolysis.

Next, we identified which of the four types of pentose residues, xylose, ribose, lyxose or arabinose, was linked to the C-3 position of subterminal GlcNAc. Because the pentose residue is acid-labile, its release from the original glycan was performed under mild acid hydrolysis conditions (Supplementary Fig. S2A). The liberated pentose was labeled with 2-aminobenzamide (2AB)^20^ and excess free-2AB subsequently removed by treatment with octanal, aldehyde reagent^21^. This procedure facilitated the detection of small amounts of reaction products to clean up the remaining unreacted labeling reagents. The released and labeled pentose was eluted from the C18 column in the same manner as the 2AB-labeled pentose (xylose, arabinose, ribose and lyxose) standard mixture. The observed retention time on HPLC and MS of the isolated peak revealed that the pentose residue linked to the free-Gn1 glycan was arabinose (Fig. 5A and Supplementary Fig. S4A).

Most arabinose found in nature corresponds to l-arabinose, which is contained in the cell walls of plants, but d-arabinose is known to be present in mycobacteria (furanose form) and trypanosomatid parasites (pyranose form)^22,23^. Therefore, to determine whether the liberated arabinose was the d- or l-enantiomer, the pentose released by mild acid hydrolysis was derivatized with l-triptophanamide (l-TrpNH_2_) via reductive amination^24^. After derivatizing the arabinose residue with l-TrpNH_2_, nonreacted excess free l-TrpNH_2_ was acetylated to increase its hydrophobicity and then removed by phenol treatment (see Materials and Methods). The sample was then analyzed alongside a standard mixture of l- and d-arabinose by RP-HPLC. This analysis unambiguously demonstrated the arabinose monosaccharide corresponded to the d-form (Fig. 5B and Supplementary Fig. S4B).

The terminal structure of this glycan, Galβ1-4(d-Ara1-3)GlcNAc, is similar to Lewis X structure. As expected, d-arabinose was cleaved by α1,3/4-l-fucosidase (Supplementary Fig. S2B). Given that α1,3/4-l-fucosidase specifically recognizes the α-anomer of l-fucose the susceptibility of the glycan to enzymatic hydrolysis by this enzyme suggested that the d-arabinose adopts a β-anomeric configuration. As a result, it was estimated that this glycan structure was Galβ1-4(d-Araβ1-3)GlcNAcβ1-4Manα1-3Manβ1-4GlcNAc-PA.

### Quantification of the free-glycan with the d-arabinose residue in urine from cancer patients

**Figure 6:**
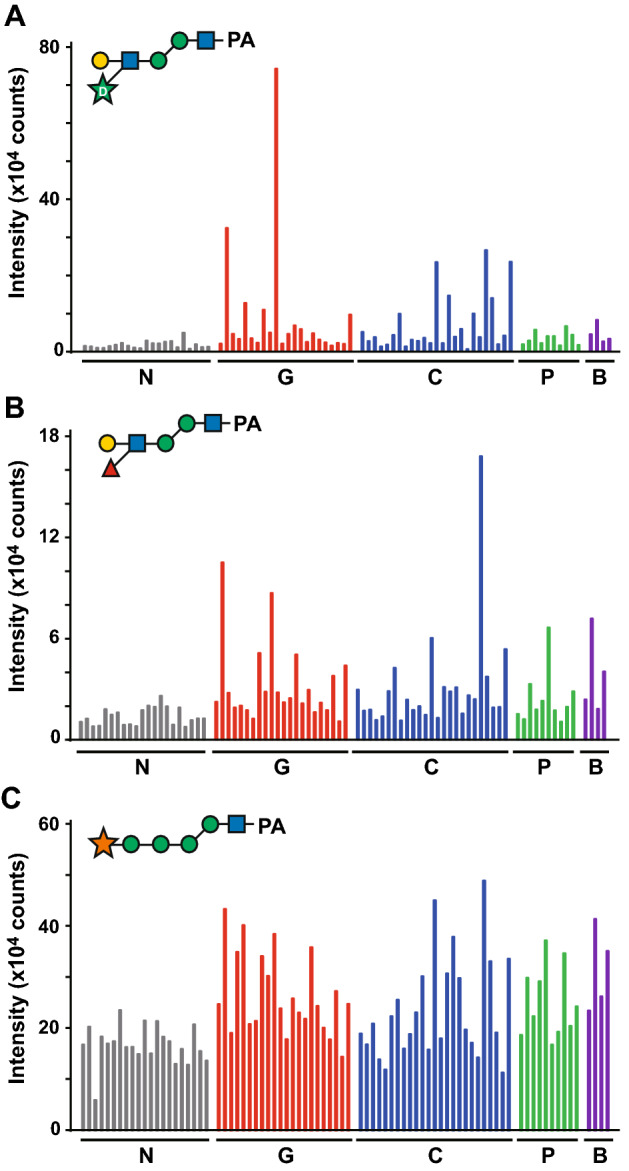
Quantification of the d-arabinose-containing glycan found in urine by SRM. The PA-glycan mixture from urine corresponding to 10 μg of creatinine was used for relative comparison of urinary free-glycan level. The peak area in the extracted ion chromatogram (XIC) of SRM transition was measured. The values of Q1 and Q3 used for measurements are shown in Supplementary Table S3. The glycan levels from urine of gastric cancer patients (G1-22), colorectal cancer patients (C1-25), pancreatic cancer patients (P1-10), bile duct cancer patients (B1-4) and normal controls (N1-21) are indicated by red, blue, green, purple and gray bars, respectively. (**A**), (**B**) and (**C**) show the level of d-arabinose-, fucose- containing *N*-glycan and xylose-containing oligomannoside, respectively.

The d-arabinose-linked glycan was found to be a major peak from the HPLC analysis of cancer patient urine. However, accurate quantification was not possible because the corresponding peak overlapped with some minor components. Accordingly, SRM was employed for the detection of this glycan contained in the urine sample. Neutral PA-glycan mixtures obtained from the urine of cancer patients and normal controls corresponding to 10 μg of creatinine were subjected to RP-HPLC/ESI–MS for simultaneous measurement of multiple glycan targets. Compared with normal subjects, patients with gastric, colorectal, pancreatic and bile duct cancers all showed relatively high urinary levels of d-arabinose-linked glycans (Fig. 6A). Indeed, several patients with gastric and colorectal cancers had particularly high levels of this glycan. All cancer specimens were confirmed to be significantly different by the Mann–Whitney test with gastric cancer *p* < 0.0001, colorectal cancer *p* < 0.0001, pancreatic cancer *p* = 0.0011 and cholangiocarcinoma *p* = 0.0014.

The arabinose-containing oligosaccharide was detected as a distinct peak in cancer patients. We reasoned that a corresponding glycan with l-fucose, rather than d-arabinose, attached to the same position might be even more abundant in vivo. Accordingly, the fucose-containing glycan, having Lewis X structure, was expected to be increased in the urine of cancer patients. However, initial screening experiments failed to detect this fucose-containing glycan. Therefore, we searched for peaks comprising Galβ1-4(Fucα1-3)GlcNAcβ1-4Manα1-3Manβ1-4GlcNAc-PA, and found that this fucose-containing glycan was included in a peak that eluted 2 min later than the peak of the arabinose-containing glycan by RP-HPLC of NP-fraction 8 (Fig. 1, gray dotted line). MS/MS analysis of this peak showed that it was composed of several glycans. The major component of this peak was an oligomannoside containing one pentose residue, which presumably corresponds to a novel glycan. The fucose-containing glycan, Galβ1-4(Fucα1-3)GlcNAcβ1-4Manα1-3Manβ1-4GlcNAc-PA, with the same elution time made up a very minor component of the peak. Another pentose-containing glycan structure was precisely analyzed by enzymatic digestion and periodate oxidation, and found to be Xylα1-3Manα1-2Manα1-2Manα1-3Manβ1-4GlcNAc-PA (Supplementary Fig. S5), with a xylose residue, not arabinose, probably attached to the terminal mannose instead of glucose. The xylose-containing oligomannoside and fucose-containing *N*-glycan were also simultaneously quantified by SRM (Fig. 6B, C). In terms of the fucose-containing glycan, a clear increase was observed in several cancer patients as seen for the arabinose-containing glycan. However, when the relative amount of arabinose- and fucose-containing glycan were compared in the same sample, the amount of fucose-containing glycans detected in cancer patients was less than that of arabinose, even though normal subjects showed comparable levels (see the scale on the Y-axis, Fig. 6A, B). Unlike the arabinose- and fucose-containing glycan, prominent elevation of the xylose-linked oligomannoside was not observed in cancer patients (Fig. 6C).

## Discussion

Our previous study focusing on acidic free-glycans identified various candidate tumor markers that are elevated in cancer patients, including novel glycan structures^16^. These findings prompted us to search for tumor markers of neutral free-glycans in urine. Unlike acidic glycans, in neutral glycans, there were fewer peaks that appeared to be predominantly elevated in cancer patient urine. The intriguing pentose-containing glycan identified in this investigation was not included in the comprehensive analysis of urine from normal subjects in our previous study^17^, because the glycan amount was either under the detection limit or deemed too low to be analyzed. Various methods were employed to obtain the precise glycan structure. Here, the identified glycan structure was a monoantennary complex-type free-*N*-glycan having a β1,4-branch on the α1,3-mannose arm with a d-arabinose residue linked to the C-3 position of subterminal GlcNAc, Galβ1-4(d-Araβ1-3)GlcNAcβ1-4Manα1-3Manβ1-4GlcNAc-PA. This is the first report to demonstrate the presence of a d-arabinose-containing glycan in human. Furthermore, this glycan level was elevated in the urine of cancer patients.

To date there are no reports on the biosynthesis of d-arabinose in human. However, it is highly likely that d-arabinose is synthesized by epimerization of other pentoses, such as ribose, which are abundant in cells. Although Neu5Gc detected in human cancer tissues has been shown to be derived from food intake^25,26^, the presence of d-arabinose in food is not reported. Thus, the possibility that d-arabinose could have arisen from food seems unlikely but cannot be completely ruled out. Alternatively, d-arabinose could be derived from the metabolites of intestinal bacteria. Although the mechanism of d-arabinosylation is unknown, it is thought that GDP-d-arabinose is converted from arabinose via the salvage pathway and utilized by fucosyltransferase as a donor substrate instead of GDP-l-fucose. Indeed, it has been reported that when d-arabinose is supplemented with media of Chinese hamster ovary cells, effective replacement (approximate 100%) of fucosylation with arabinosylation occurred in the *N*-glycan of immunoglobulin^27^. GDP-arabinose was found to be less effective than GDP-fucose (5.9%) but sufficiently reactive as a donor of α1,4-fucosyltransferase^28^. Although the efficacy of GDP-arabinose as a donor substrate to α1,3-fucosyltransferase has not been investigated, it is likely to be effective. The upregulation of α1,3-fucosyltransferase, which forms the Lewis X structure, has been reported in various cancers^29^. This elevation in the level of α1,3-fucosyltransferase may be involved, at least partially, in the increase of d-arabinose-containing free-*N*-glycan in the urine of cancer patients. Indeed, the fucosylation site of *N*-glycans found in plasma proteins is at the C-3 position of GlcNAc in the β1,4-branch on α1,3-mannose arm^30,31^, which corresponds with the linkage position of d-arabinose.

The d-arabinose-containing free-*N*-glycan was detected as a distinct peak in the RP chromatogram of cancer patients. By contrast, the fucose-containing oligosaccharide with a Lewis X structure on the same backbone, Galβ1-4(l-Fucα1-3)GlcNAcβ1-4Manα1-3Manβ1-4GlcNAc-PA, was not detected as an obvious peak. Indeed, this glycan overlaps the xylose-containing glycan in RP-chromatograms of NP-fraction 8 and is present at a negligible level. SRM revealed that the level of this fucose-containing glycan in cancer patients seemed to be markedly elevated by comparison to normal controls. However, when comparing the level of the fucose-containing glycan and the d-arabinose-containing glycan in the same sample, especially in cancer patients, the level of the d-arabinose-containing glycan is higher than that of the fucose-containing glycan. It is not obvious why the level of the fucose-containing glycan is lower than that of the d-arabinose-containing glycan. The arabinose-containing *N*-glycan may be less easily degraded than the fucose-containing *N*-glycan. Although human α-l-fucosidase can recognize both β-d-arabinose and α-l-fucose as substrate, the enzyme has a much lower specific activity for β-d-arabinose than for α-l-fucose i.e., the Km value is about 30-fold higher than for α-l-fucose^32^. In the case of mature *N*-glycans attached to proteins, the amount of fucose-containing glycan would be much higher than that of arabinose. However, during the degradation process, such as ERAD, most of the fucose-containing glycans are cleaved by fucosidase, followed by a series of degrading enzymes, while many of the arabinose-containing glycans may escape degradation by fucosidase digestion and remain intact. Therefore, it is possible that the relative amount of each glycan is reversed in the final urinary samples.

In summary, this is the first study to report the identification and characterization of a d-arabinose-containing oligosaccharide in human. Intriguingly, measurement of glycan levels using SRM showed that the *N*-glycan with d-arabinose was markedly elevated in the urine of cancer patients. Whether or not the detection of d-arabinosylated *N*-glycans in urine samples from cancer patients can be utilized as a marker for cancer diagnosis requires further investigation.

## Methods

### Urine samples

Urine samples were obtained from cancer-free control volunteers (*n* = 21; male 15, female 6, mean age 63.2 years), patients with gastric cancer (*n* = 22; male 11, female 11, mean age 65.3 years), colorectal cancer (*n* = 25; male 8, female 17, mean age 59.8 years), pancreatic cancer (*n* = 10; male 6, female 4, mean age 62.7 years) and bile duct cancer (*n* = 4; male 2, female 2, mean age 68.0 years) at the Osaka International Cancer Institute. All cancer patients are not administered anticancer drugs. Clinical information is shown in Supplementary Table S1. This study was approved by the Local Ethics Committee of Osaka International Cancer Institute. Informed consent was obtained from all the volunteers and cancer patients. All experiments were conducted in accordance with relevant guidelines and regulations.

### Isolation of free-glycans from urine samples

Urinary free-glycans were prepared as previously reported^16^. Briefly, the volume of the urine specimens used for sample preparation were corrected to normalize for creatinine concentration. Free-glycans were extracted from the urine samples equivalent to 400 µg creatinine by chromatography using Dowex 50W-X8 (H^+^ form, 100–200 mesh, FUJIFILM Wako Pure Chemical, Osaka, Japan) followed by a graphite carbon cartridge (InertSepGC 300 mg; GL Science, Tokyo, Japan).

### Preparation and separation of PA-glycans

Experimental procedures, such as preparation and separation of PA-glycans, were performed as previously reported^11,16^. The reducing ends of urinary free-glycans were labeled with 2-aminopyridine by reductive amination^33,34^. Excess reagents were subsequently removed by phenol/chloroform extraction and cation exchange chromatography. PA-glycans were then loaded onto a graphite carbon cartridge (InertSepGC 150 mg; GL Science)^35^. To collect the neutral fractions, weak anion exchange HPLC was performed on a TSKgel DEAE-5PW column (10 μm, 7.5 × 75 mm; Tosoh, Tokyo, Japan). The following experimental procedures, including NP-HPLC and RP-HPLC have been reported previously^36^. The above HPLC conditions are described in Supplementary Table S2 according to the Minimum Information Required for A Glycomics Experiment (MIRAGE) guidelines.

### Glycosidase digestion

*N*-acetyl-β-d-glucosaminidase S from *Streptococcus pneumoniae* (New England Biolabs, Ipswich, MA, USA) and α1,3/4-l-fucosidase from *Streptomyces sp. 142* (Takara Bio, Shiga, Japan) were used in 1 × GlycoBuffer 1 (50 mM sodium acetate and 5 mM CaCl_2_ pH 5.5; New England Biolabs) at 37 °C overnight. β4-d-Galactosidase from *S. pneumoniae* (Agilent, Santa Clara, CA, USA) was incubated in sodium citrate buffer (pH 6.0) at 37 °C. Sodium acetate (100 mM pH 5.0) with 2 mM zinc chloride was used as the buffer for α1, 2/3/6-mannosidase from Jack Bean (Agilent). Sodium phosphate (100 mM pH 6.8) was used as the buffer for α-xylosidase from *E. coli* (Megazyme, Bray, Ireland). Purified *N*-glycans released from α1-acid glycoprotein in human plasma (Sigma-Aldrich, St Louis, Mo, USA) with Endo F3 (New England Biolabs) were labeled with 2-aminopyridine for use as standard glycans. The standard for fucosylated *N*-glycan with an α1,3-mannose arm extended β4-branching lactosamine was prepared by enzymatic digestion of triantennary *N*-glycan with sialidase, galactosidase, hexosaminidase and mannosidase, without fucosidase treatment.

### Periodate oxidation and reduction of PA-glycans

Vacuum dried PA-glycans were treated with 80 mM sodium periodate in 50 mM sodium acetate buffer (pH 4.0) at 4 °C for 2 days in the dark. The reaction was stopped by addition of 50% ethylene glycol at room temperature for 1 h, followed by reduction with 200 mM sodium borohydride in 100 mM sodium borate buffer (pH 8.0) for 2 h^37,38^. The resulting glycans were desalted on a graphite carbon cartridge and analyzed by ESI–MS and MS^n^.

### Permethylation of PA-glycans

Vacuum dried PA-glycans were dissolved in NaOH/dimethyl sulfoxide added 1% distilled water slurry^39,40^. After vigorous mixing, iodomethane was added into slurry mixture. The reaction mixtures were vortexed for 20 min before adding ice-cold 2.5% acetic acid. The remaining iodomethane was removed by N_2_-gas bubbling. Permethylated glycans were purified using Oasis PRiME HLB (Waters Co., Milford, MA, USA) and lyophilized. The lyophilized material was then redissolved in distilled water containing 10% acetonitrile, 10% *N,N*-dimethylformamide and 0.05% acetic acid.

### Preparation and separation of 2AB-monosaccharide

Pentose monosaccharides were obtained from PA-labeled free-*N*-glycan analyte by partial acid hydrolysis^41^ or α1,3/4-fucosidase treatment. Pentose residues were released from PA-labeled free-*N*-glycan by mild acid hydrolysis with 0.1 M TFA at 80 °C for 3 h. Monosaccharides liberated from the original glycan structure by partial acid hydrolysis were loaded onto a graphite carbon cartridge (GL Science) to facilitate separation from PA-labeled oligosaccharides, and the flowthrough was collected. The lyophilized monosaccharides or standards were incubated with 2.5 μmol 2-aminobenzamide (Sigma-Aldrich) and 600 nmol picoline borate (Junsei Chemical Co. Ltd., Tokyo, Japan) in 5 μL methanol/acetic acid/DDW (v/v/v, 85:10:5) at 40 °C for 3 h^20^. The 2AB labeled monosaccharides were evaporated and then finally dissolved in 1 mL of distilled water. Unreacted excess free-2AB after the labeling reaction were removed by the addition of octanal to the 2AB labeled solution followed by vigorous vortexing. The aqueous layer was separated by centrifugation, followed by the addition of chloroform and vigorous mixing. The aqueous layer was separated by centrifugation and filtered^21^. The 2AB labeled monosaccharides were separated using a Shim-pack Scepter C18-120 (2.1 × 150 mm, 3 μm; Shimadzu, Kyoto, Japan) at 55 °C. Solvent A (0.1% formic acid) and solvent B (0.1% formic acid in 50% acetonitrile) were used at a flow rate of 0.2 ml/min. The column was equilibrated with 2% solvent B and then solvent B was linearly increased to 15% in 40 min. The eluate was monitored with excitation and emission wavelengths of 330 and 420 nm, respectively (Supplementary Table S2). All standard monosaccharides were purchased from TCI (Tokyo Chemical Industry Co. Ltd., Tokyo, Japan), and labeled with 2AB.

### Derivatization and separation of monosaccharide with l-TrpNH_2_

Arabinose monosaccharides were obtained by partial acid hydrolysis, as mentioned earlier for 2AB labeling^41^. Derivatization of arabinose monosaccharides was performed using the method described by Shou et al.^24^. Briefly, monosaccharides dissolved in 10 μL distilled water were incubated with 30 μL of 400 mM l-TrpNH_2_ in methanol/100 mM tetraborate (v/v, 1:2) and 10 μL of 2 M dimethylamine borate at 40 °C for 4 h. Then, 50 μL each of acetic anhydride, 100 mM tetraborate and 1 M NaCl were added to the reaction mixtures followed by vortexing. The free amino group of unreacted l-TrpNH_2_ was acetylated by incubation for 30 min. Additionally, 50 μL each of 10% formic acid and distilled water were added to the reaction mixtures, which were then subjected to two consecutive phenol–chloroform extractions for the removal of unreacted excess l-TrpNH_2_. The recovered aqueous solution was purified by loading onto a graphite carbon cartridge (GL Science). The l-TrpNH_2_ derivatives were separated using a Shim-pack Scepter C18-120 (2.1 × 150 mm, 5 μm; Shimadzu) at 40 °C. Solvent A (2.5 mM *n*-butylboronic acid, 100 mM phosphate, 5% acetonitrile, pH 7.7) and solvent B (20% acetonitrile) were used for the linear gradient elution at a flow rate of 0.2 ml/min^24^. The eluate was monitored with excitation and emission wavelengths of 280 and 350 nm, respectively (Supplementary Table S2). d- and l-arabinoses purchased from TCI were derivatized with l-TrpNH_2_, and used as standards.

### Mass spectrometry

Mass spectrometry for glycan structural analysis was set up as previously reported^16^. For the detection of monosaccharide derivatives, the ESI–MS on a LTQ XL linear ion trap mass spectrometer (Thermo Scientific, San Jose, CA, USA) was connected to an LC-20AD HPLC system (Shimadzu). The separation of both 2AB- and l-TrpNH_2_-monosaccharides derivatives by liquid chromatography was performed as mentioned earlier for the separation of 2AB-monosaccharides (Supplementary Table S2). MS spray voltage was set to 3 kV in positive ion mode. The temperature of the ion source was maintained at 250 °C. The following parameters were used: temperature of the capillary, 300 °C; sheath gas, 40 units; tube lens voltage, 100 V; range for a full MS scan, 250–400.

### SRM for quantification of glycans

A 4500 QTRAP hybrid triple quadrupole/linear ion trap mass spectrometer (AB SCIEX, Framingham, MA, USA) connected to LC-20AD HPLC system (Shimadzu) was used for the quantification of glycan by SRM. The urinary neutral PA-glycan mixture corresponding to 10 μg of creatinine were separated by Shim-pack Scepter C18-120 (2.1 × 150 mm, 3 μm; Shimadzu) at 35 °C. The solvents, 10 mM acetic acid with triethylamine (pH 6.0) (A) and 10 mM acetic acid with triethylamine (pH 6.0) in 50% acetonitrile (B), were used for gradient elution. The eluate was analyzed by electrospray ionization with post-column addition of acetonitrile (Supplementary Table S2). Quality control was prepared by pooling equal volumes of all urine PA-glycan samples, and analyzed every 10 runs for analysis monitoring. 50 fmol of C^13^-panose was spiked into each glycan sample as an internal standard. Data acquisition was performed with an ion spray voltage of 5 kV, curtain gas of 30 psi, collision gas of 9 psi, nebulizer gas (GS1) of 65 psi, turbo gas (GS2) of 55 psi, an interface heater temperature of 500 °C and dwell time of 50 ms. Two SRM transitions were monitored and acquired at low resolution both in the first and third quadrupoles (Q1 and Q3). The values of Q1 and Q3 are shown in Supplementary Table S3. SRM data acquired on the 4500 QTRAP were analyzed by SCIEX OS (AB SCIEX). The peak area in the extracted ion chromatogram (XIC) of each SRM transition was measured.

## Supplementary Information

Supplementary Information.

## Data Availability

All data generated or analyzed during the study are included either in this published article and its supplementary information file or can be made available from the corresponding author on reasonable request or from UniCarb-DR (https://unicarb-dr.glycosmos.org/references).

## References

[1] Varki A (2017). Biological roles of glycans. Glycobiology.

[2] Parodi, A., Cummings, R. D. & Aebi, M. in *Essentials of glycobiology* (eds A. Varki *et al.*) 503–511 (Cold Spring Harbor Laboratory Press Copyright 2015–2017 by The Consortium of Glycobiology Editors, La Jolla, California. All rights reserved., 2015).

[3] Moremen KW, Tiemeyer M, Nairn AV (2012). Vertebrate protein glycosylation: diversity, synthesis and function. Nat. Rev. Mol. Cell Biol..

[4] Ohashi S, Iwai K, Mega T, Hase S (1999). Quantitation and isomeric structure analysis of free oligosaccharides present in the cytosol fraction of mouse liver: detection of a free disialobiantennary oligosaccharide and glucosylated oligomannosides. J. Biochem..

[5] Ishizuka A (2008). Accumulation of free complex-type N-glycans in MKN7 and MKN45 stomach cancer cells. Biochem. J..

[6] Iwatsuka K (2013). Free glycans derived from glycoproteins present in human sera. J. Chromatogr. B Anal. Technol. Biomed. Life Sci..

[7] Peelen GO, de Jong JG, Wevers RA (1994). HPLC analysis of oligosaccharides in urine from oligosaccharidosis patients. Clin. Chem..

[8] Bruggink C, Poorthuis BJ, Deelder AM, Wuhrer M (2012). Analysis of urinary oligosaccharides in lysosomal storage disorders by capillary high-performance anion-exchange chromatography-mass spectrometry. Anal. Bioanal. Chem..

[9] Xia B (2013). Oligosaccharide analysis in urine by maldi-tof mass spectrometry for the diagnosis of lysosomal storage diseases. Clin. Chem..

[10] Yabu M, Korekane H, Miyamoto Y (2014). Precise structural analysis of O-linked oligosaccharides in human serum. Glycobiology.

[11] Tanaka-Okamoto M (2016). Elevation of CA19-9-related novel marker, core 1 sialyl lewis A, in sera of adenocarcinoma patients verified by a SRM-based method. J. Proteome Res..

[12] Tanaka-Okamoto M (2017). Various sulfated carbohydrate tumor marker candidates identified by focused glycomic analyses. Glycobiology.

[13] Tanaka-Okamoto M (2018). Identification of internally sialylated carbohydrate tumor marker candidates, including Sda/CAD antigens, by focused glycomic analyses utilizing the substrate specificity of neuraminidase. Glycobiology.

[14] Yabu M (2013). Accumulation of free Neu5Ac-containing complex-type N-glycans in human pancreatic cancers. Glycoconj. J..

[15] Yabu M (2013). Occurrence of free deaminoneuraminic acid (KDN)-containing complex-type N-glycans in human prostate cancers. Glycobiology.

[16] Hanzawa K (2021). Investigation of acidic free-glycans in urine and their alteration in cancer. Glycobiology.

[17] Tanaka-Okamoto M, Hanzawa K, Murakami H, Mukai M, Miyamoto Y (2021). Identification of β1-3 galactosylglucose-core free-glycans in human urine. Anal. Biochem..

[18] Harvey DJ (2002). "Internal residue loss": rearrangements occurring during the fragmentation of carbohydrates derivatized at the reducing terminus. Anal. Chem..

[19] Hecht ES, Loziuk PL, Muddiman DC (2017). Xylose migration during tandem mass spectrometry of N-linked glycans. J. Am. Soc. Mass Spectr..

[20] Fang J, Qin G, Ma J, She YM (2015). Quantification of plant cell wall monosaccharides by reversed-phase liquid chromatography with 2-aminobenzamide pre-column derivatization and a non-toxic reducing reagent 2-picoline borane. J. Chromatogr. A.

[21] Chu AA, Saati AE, Scarcelli JJ, Cornell RJ, Porter TJ (2018). Reactivity-driven cleanup of 2-Aminobenzamide derivatized oligosaccharides. Anal. Biochem..

[22] Chatterjee D, Bozic CM, McNeil M, Brennan PJ (1991). Structural features of the arabinan component of the lipoarabinomannan of *Mycobacterium tuberculosis*. J. Biol. Chem..

[23] McConville MJ, Thomas-Oates JE, Ferguson MA, Homans SW (1990). Structure of the lipophosphoglycan from Leishmania major. J. Biol. Chem..

[24] Shou M (2015). Simultaneous enantioseparation of aldohexoses and aldopentoses derivatized with L-tryptophanamide by reversed phase HPLC using butylboronic acid as a complexation reagent of monosaccharides. Chirality.

[25] Varki A (2010). Colloquium paper: uniquely human evolution of sialic acid genetics and biology. Proc. Natl. Acad. Sci. USA.

[26] Bardor M, Nguyen DH, Diaz S, Varki A (2005). Mechanism of uptake and incorporation of the non-human sialic acid N-glycolylneuraminic acid into human cells. J. Biol. Chem..

[27] Hossler P (2017). Arabinosylation of recombinant human immunoglobulin-based protein therapeutics. MAbs.

[28] Gokhale UB, Hindsgaul O, Palcic MM (1990). Chemical synthesis of GDP-fucose analogs and their utilization by the Lewis *A(1→4) fucosyltransferase. Can. J. Chem..

[29] Blanas A, Sahasrabudhe NM, Rodríguez E, van Kooyk Y, van Vliet SJ (2018). Fucosylated antigens in cancer: an alliance toward tumor progression, metastasis, and resistance to chemotherapy. Front. Oncol..

[30] Endo M (1982). The structures and microheterogeneity of the carbohydrate chains of human plasma ceruloplasmin: a study employing 500-MHz 1H-NMR spectroscopy. J. Biol. Chem..

[31] Kolarich D, Weber A, Turecek PL, Schwarz HP, Altmann F (2006). Comprehensive glyco-proteomic analysis of human alpha1-antitrypsin and its charge isoforms. Proteomics.

[32] Wiederschain G, Beyer EM, Klyashchitsky BA, Shashkov AS (1981). Specificity patterns of different types of human fucosidase. Recognition of a certain region of the pyranose ring in sugars by the enzymes. Biochem. Biophys. Acta..

[33] Hase S, Ikenaka T, Matsushima Y (1978). Structure analyses of oligosaccharides by tagging of the reducing end sugars with a fluorescent compound. Biochem. Biophys. Res. Commun..

[34] Kuraya N, Hase S (1992). Release of O-linked sugar chains from glycoproteins with anhydrous hydrazine and pyridylamination of the sugar chains with improved reaction conditions. J. Biochem..

[35] Natsuka S, Hirohata Y, Nakakita S, Sumiyoshi W, Hase S (2011). Structural analysis of N-glycans of the planarian *Dugesia japonica*. FEBS J..

[36] Misonou Y (2009). Comprehensive clinico-glycomic study of 16 colorectal cancer specimens: elucidation of aberrant glycosylation and its mechanistic causes in colorectal cancer cells. J. Proteome Res..

[37] Minamida S (1996). Detection of UDP-D-xylose: alpha-D-xyloside alpha 1–>3xylosyltransferase activity in human hepatoma cell line HepG2. J. Biochem..

[38] Irimura T, Tsuji T, Tagami S, Yamamoto K, Osawa T (1981). Structure of a complex-type sugar chain of human glycophorin A. Biochemistry.

[39] Hakomori S (1964). A rapid permethylation of glycolipid, and polysaccharide catalyzed by methylsulfinyl carbanion in dimethyl sulfoxide. J. Biochem..

[40] Ciucanu I, Costello CE (2003). Elimination of oxidative degradation during the per-O-methylation of carbohydrates. J. Am. Chem. Soc..

[41] Makino Y, Omichi K, Hase S (1998). Analysis of oligosaccharide structures from the reducing end terminal by combining partial acid hydrolysis and a two-dimensional sugar map. Anal. Biochem..

