# Supplementary material for: Occurrence of a d-arabinose-containing complex-type free-N-glycan in the urine of cancer patients
Source: Sci Rep. 2022 Mar 22;12:4889. doi: 10.1038/s41598-022-08790-0 (PMC8941101; doi:10.1038/s41598-022-08790-0)
Supplement: Supplementary file 1 — Supplementary Information. [file 41598_2022_8790_MOESM1_ESM.docx]

**Supplementary Tables and Figures**

**Occurrence of a d-arabinose-containing complex-type free-*N*-glycan in the urine of cancer patients**

**Miki Tanaka-Okamoto, Ken Hanzawa, Hiroko Murakami, Mikio Mukai, Hidenori Takahashi,** **Takeshi Omori,** **Kenji Ikezawa,** **Kazuyoshi Ohkawa, Masayuki Ohue and Yasuhide Miyamoto**

**Supplementary Table S1.**

Clinical information for normal subjects and cancer patients

| Sample ID. | Sex | Age | ABO blood group | Urine creatinine (mg/dL) | CA19-9 (U/mL) | CEA (ng/mL) |
| --- | --- | --- | --- | --- | --- | --- |
| N1 | M | 64 | O | 61.6 | <2 | nd |
| N2 | M | 69 | O | 175 | 7 | nd |
| N3 | M | 69 | A | 59.8 | 2 | nd |
| N4 | M | 77 | A | 190.6 | 4 | nd |
| N5 | M | 68 | O | 116.7 | 2 | nd |
| N6 | F | 53 | AB | 140.1 | 3 | nd |
| N7 | F | 70 | O | 77.9 | 3 | nd |
| N8 | M | 75 | A | 84 | 14 | nd |
| N9 | M | 81 | A | 83.1 | 2 | nd |
| N10 | M | 50 | B | 84.1 | 12 | nd |
| N11 | F | 75 | B | 58.8 | 2 | nd |
| N12 | M | 69 | A | 105.1 | 4 | nd |
| N13 | F | 33 | A | 72.9 | 5 | nd |
| N14 | F | 56 | O | 115.8 | 2 | nd |
| N15 | M | 81 | AB | 80.6 | 4 | nd |
| N16 | M | 50 | A | 281.4 | 8 | nd |
| N17 | M | 71 | A | 43.8 | 12 | nd |
| N18 | M | 42 | A | 94.2 | 3 | nd |
| N19 | F | 61 | B | 77.2 | 5 | nd |
| N20 | M | 66 | A | 126.9 | 4 | nd |
| N21 | M | 48 | A | 148.4 | 3 | nd |
| G1 | F | 61 | B | 32.1 | 4 | 1.1 |
| G2 | F | 65 | A | 384.7 | 67 | 1 |
| G3 | M | 65 | B | 115.3 | 206 | 60 |
| G4 | M | 78 | AB | 160.6 | 568 | 6.2 |
| G5 | F | 70 | B | 319.4 | <2 | 1.2 |
| G6 | M | 64 | O | 190.4 | 4 | 7.5 |
| G7 | M | 72 | A | 115.7 | 76 | 1.7 |
| G8 | M | 74 | A | 186.4 | 3 | 1.1 |
| G9 | M | 59 | A | 28.6 | 1063 | 27.4 |
| G10 | F | 67 | O | 162.9 | 2 | 1.2 |
| G11 | F | 62 | O | 187.1 | 16 | 549.9 |
| G12 | M | 60 | A | 254.3 | 1187 | 4.5 |
| G13 | F | 74 | O | 52.6 | 46 | 17.7 |
| G14 | F | 34 | O | 243.2 | 68 | 1.6 |
| G15 | M | 61 | O | 107.5 | 18 | 0.7 |
| G16 | F | 76 | A | 345.7 | 4 | 2.1 |
| G17 | F | 71 | A | 215.7 | 5 | 0.7 |
| G18 | F | 56 | A | 46.2 | 5 | 1.6 |
| G19 | M | 66 | B | 268.5 | 6 | 2.1 |
| G20 | F | 69 | O | 188.8 | 474 | 304.1 |
| G21 | M | 71 | O | 230.5 | 13 | 10.6 |
| G22 | M | 61 | AB | 150.2 | 14 | 13.4 |
| C1 | F | 56 | O | 13.7 | 41804 | 1584.9 |
| C2 | M | 58 | B | 54.6 | <2 | 4.2 |
| C3 | F | 62 | A | 84.2 | 252 | 39.4 |
| C4 | F | 25 | A | 133.2 | 7732 | 6.2 |
| C5 | M | 47 | B | 215.6 | 30 | 1.7 |
| C6 | F | 51 | A | 207.1 | 59 | 56 |
| C7 | F | 82 | A | 82.8 | 27 | 4.9 |
| C8 | M | 38 | A | 65.5 | 10 | 19.2 |
| C9 | M | 71 | O | 165.8 | 16050 | 83.9 |
| C10 | F | 74 | O | 24.5 | 11 | 6 |
| C11 | F | 70 | B | 106.3 | 50 | 35.5 |
| C12 | F | 45 | A | 37.3 | 459 | 854 |
| C13 | M | 70 | A | 198.2 | 14038 | 126.4 |
| C14 | F | 68 | AB | 351.0 | 307 | 85.1 |
| C15 | F | 71 | A | 182.6 | >100000 | 3472.6 |
| C16 | F | 51 | A | 104.8 | 54545 | 1129.5 |
| C17 | F | 64 | A | 34.7 | 4 | 19.1 |
| C18 | F | 67 | A | 96.1 | 4 | 1.6 |
| C19 | M | 65 | O | 201.6 | 294 | 13.1 |
| C20 | M | 55 | O | 357.0 | 1935 | 233.1 |
| C21 | F | 63 |  | 145.2 | 8 | 7.3 |
| C22 | F | 73 | A | 128.1 | 9 | 68.8 |
| C23 | F | 61 | B | 75.1 | 363 | 168.1 |
| C24 | M | 55 | AB | 269.5 | 2 | 3.3 |
| C25 | F | 53 | O | 34.5 | 328 | 132.8 |
| P1 | M | 48 | A | 69.4 | 3311 | 3.7 |
| P2 | F | 58 | B | 180.5 | >100000 | 560.2 |
| P3 | M | 68 | A | 439.5 | >100000 | 220.5 |
| P4 | F | 50 | A | 50.5 | 16421 | 11.5 |
| P5 | F | 66 | B | 159.5 | <2 | 3 |
| P6 | M | 62 | A | 270.1 | 46597 | 4.8 |
| P7 | M | 72 | A | 51.5 | 20124 | 13.1 |
| P8 | F | 62 | A | 116.2 | 371 | 7.3 |
| P9 | M | 64 | O | 187.7 | >100000 | 162.9 |
| P10 | M | 77 | A | 59.7 | >100000 | 42 |
| B1 | F | 55 | O | 74.6 | 29046 | 206 |
| B2 | M | 65 | AB | 363.8 | 32678 | 1673.1 |
| B3 | F | 78 | O | 45.4 | >100000 | 164.4 |
| B4 | M | 74 | O | 153.4 | 81803 | 156.2 |

N, G, C, P and B of sample IDs represent normal subjects and gastric, colorectal, pancreatic and bile duct cancer patients, respectively. Cut-off values of CA19-9 and CEA are 37 U/mL and 5 ng/mL, respectively. F, female; M, male. nd indicates not determined.

**Supplementary Table S2.**

Detailed conditions of the HPLC used in this study.

| (1) Anion-exchange HPLC | | |
| --- | --- | --- |
| Solvent | A: | ~0.3 mM aqueous ammonia (28% NH_3_ diluted with water into 1/500,000) |
| Solvent | B: | 1 M acetic acid adjusted to pH9.0 with aqueous NH_3_ |
| Column | : | TSKgel DEAE-5PW (10 μm, 7.5 × 75 mm; Tosoh) |
| Column | temp. (°C): | 30 |
| Flow rate | (mL/min): | 0.8 |
| Fluorescence | Ex/Em (nm) | : 310/380 |
|  |  |  |
| Gradient |  |  |
| Time (min) | % sol. B | 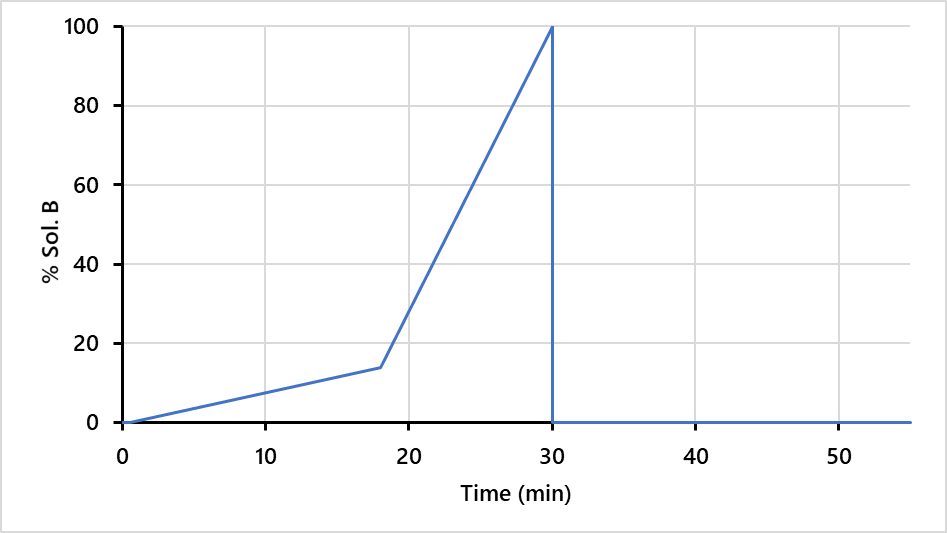 |
| 0.00 | 0.0 |  |
| 0.50 | 0.0 |  |
| 18.00 | 14.0 |  |
| 30.00 | 100.0 |  |
| 30.01 | 0.0 |  |
| 55.00 | 0.0 |  |
|  |  |  |
| (2) Normal phase HPLC | | |
| Solvent | A: | 5:1 (v/v), Acetonitrile / 0.5M acetic acid+10% (v/v) acetonitrile,  adjusted to pH7.3 with triethylamine |
| Solvent | B: | 4:5 (v/v), Acetonitrile / 0.5M acetic acid+10% (v/v) acetonitrile,  adjusted to pH7.3 with triethylamine |
| Column | : | TSKgel Amide-80 (5 μm, 2 × 250 mm; Tosoh) |
| Column | temp. (°C): | 40 |
| Flow rate | (mL/min): | 0.2 |
| Fluorescence | Ex/Em(nm): | 310/380 |
|  |  |  |
| Gradient |  | |
| Time (min) | % sol. B | 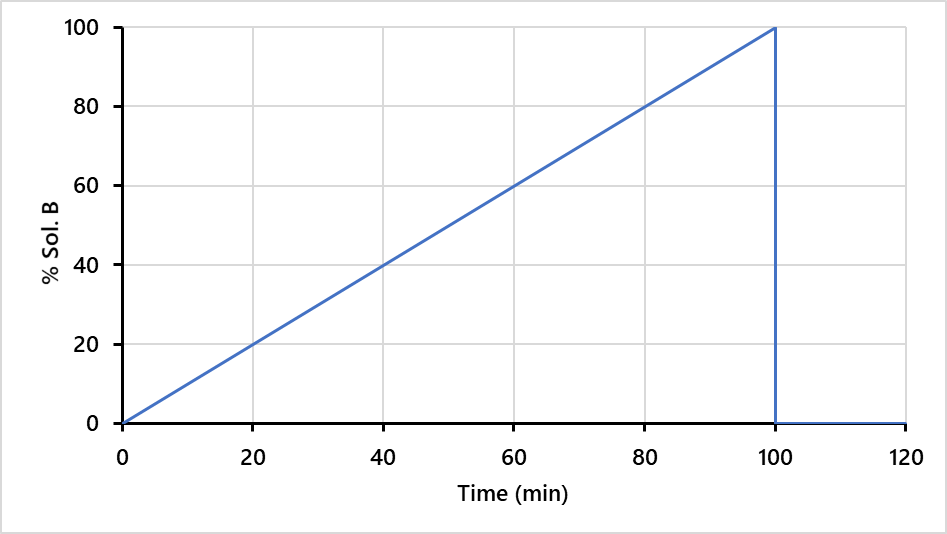 |
| 0.00 | 0.0 |  |
| 100.00 | 100.0 |  |
| 100.01 | 0.0 |  |
| 120.00 | 0.0 |  |
|  |  |  |
|  |  |  |
| (3) Reversed phase HPLC for PA-glycan | | |
| Solvent | A: | 9:1 (v/v), Water / 0.5M acetic acid, adjusted to pH6.0 with triethylamine |
| Solvent | B: | 7:2:1 (v/v), Water / Acetonitrile / 0.5M acetic acid, adjusted to pH6.0 with triethylamine |
| Column | : | TSKgel ODS-80Ts QA (5 μm, 2 × 150 mm; Tosoh) |
| Column | temp. (°C): | 30 |
| Flow rate | (mL/min): | 0.2 |
| Fluorescence | Ex/Em (nm) | 315/400 |
|  |  |  |
| Gradient |  | 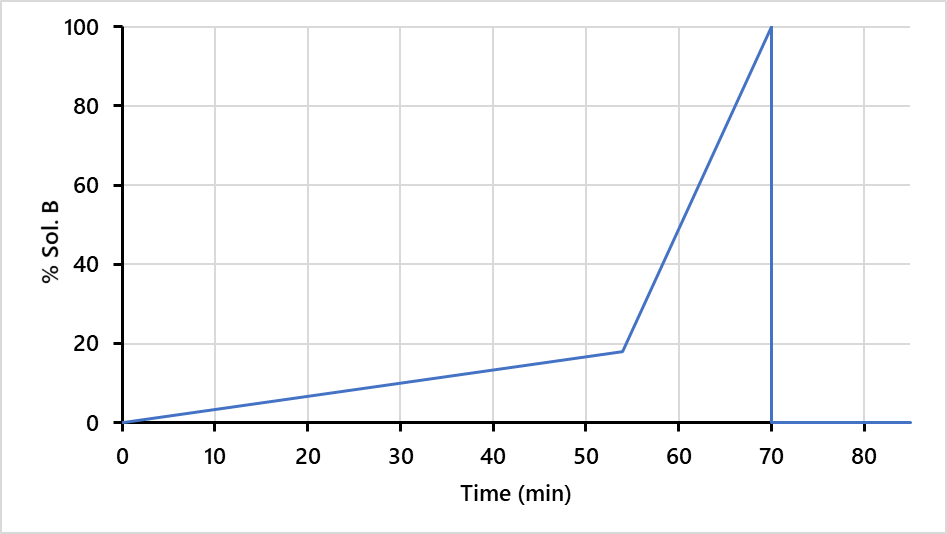 |
| Time (min) | % sol. B |  |
| 0.00 | 0.0 |  |
| 54.00 | 18.0 |  |
| 70.00 | 100 |  |
| 70.01 | 0.0 |  |
| 85.00 | 0.0 |  |
|  |  |  |
|  |  |  |

**Supplementary Table S2. (continued)**

| (4) Reversed phase HPLC for 2AB-monosaccharide | | |
| --- | --- | --- |
| Solvent | A: | 0.1% (v/v) Formic acid / Water |
| Solvent | B: | 0.1% (v/v) Formic acid / 50% (v/v) Acetonitrile in Water |
| Column | : | Shim-pack Scepter C18-120 (3 μm, 2.1 × 150 mm; Shimadzu) |
| Column | temp. (°C): | 55 |
| Flow rate | (mL/min): | 0.2 |
| Fluorescence | Ex/Em (nm) | 330/420 |
|  |  |  |
| Gradient |  | 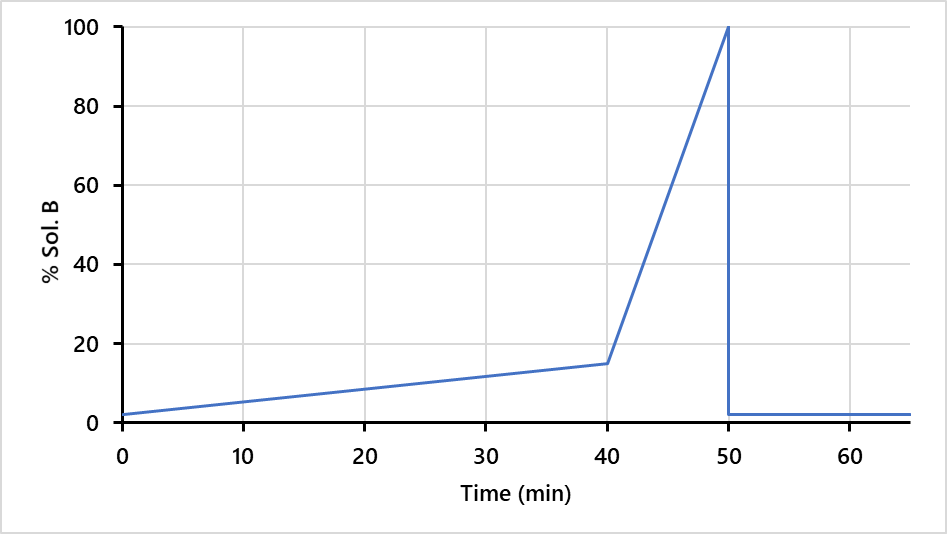 |
| Time (min) | % sol. B |  |
| 0.00 | 2.0 |  |
| 40.00 | 15.0 |  |
| 50.00 | 100.0 |  |
| 50.01 | 2.0 |  |
| 65.00 | 2.0 |  |
|  |  |  |
| (5) Reversed phase HPLC for TrpNH_2_-monosaccharide | | |
| Solvent | A: | 2.5mM Butylboronic acid, 5% Acetonitrile 100mM phosphate in Water, adjusted to pH7.7 with NH_3_ |
| Solvent | B: | 20% (v/v) Acetonitrile in Water |
| Column | : | Shim-pack Scepter C18-120 (5 μm, 2.1 × 150 mm; Shimadzu) |
| Column | temp. (°C): | 40 |
| Flow rate | (mL/min): | 0.2 |
| Fluorescence | Ex/Em (nm) | 280/350 |
|  |  |  |
| Gradient |  | 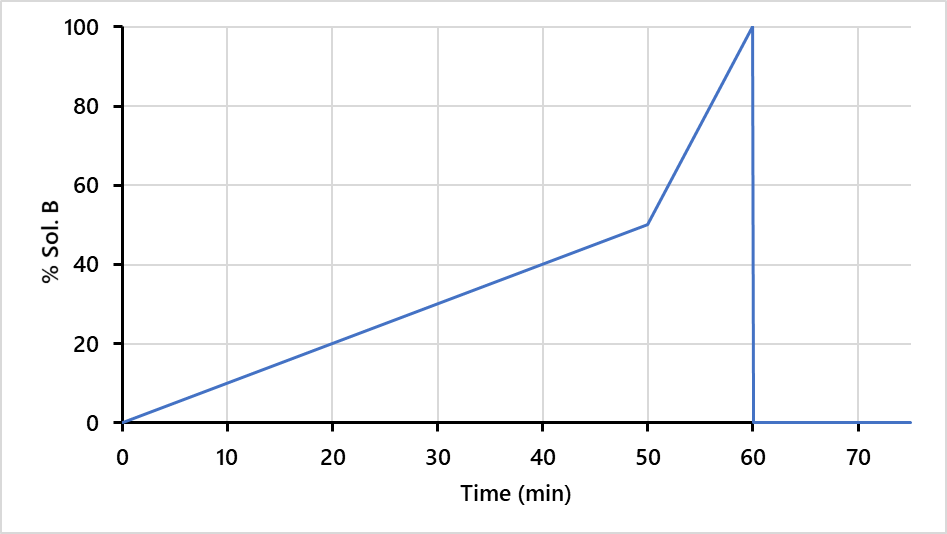 |
| Time (min) | % sol. B |  |
| 0.00 | 0.0 |  |
| 50.00 | 50.0 |  |
| 60.00 | 100.0 |  |
| 60.01 | 0.0 |  |
| 75.00 | 0.0 |  |
|  |  |  |
| (6) Reversed phase HPLC for MS of monosaccharide | | |
| Solvent | A: | 0.1% (v/v) Formic acid in Water |
| Solvent | B: | 0.1% (v/v) Formic acid in 50% (v/v) Acetonitrile in Water |
| Column | : | Shim-pack Scepter C18-120 (3 μm, 2.1 × 150 mm; Shimadzu) |
| Column | temp. (°C): | 55°C |
| Flow rate | (mL/min): | 0.2 |
|  |  |  |
| Gradient |  |  |
| Time (min) | % sol. B | 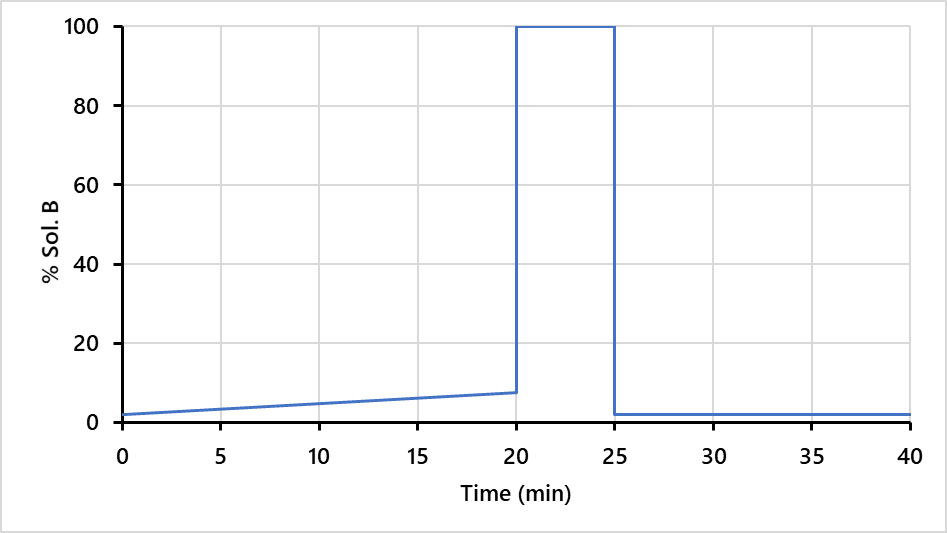 |
| 0.00 | 2.0 |  |
| 20.00 | 7.5 |  |
| 20.01 | 100.0 |  |
| 25.00 | 100.0 |  |
| 25.01 | 2.0 |  |
| 40.00 | 2.0 |  |
|  |  |  |
|  |  |  |
|  |  |  |

**Supplementary Table S2. (continued)**

| (7) Reversed phase HPLC for MS of glycan structural analysis | | | |
| --- | --- | --- | --- |
| Solvent | A: | 99:1 (v/v), Water / 0.5M acetic acid, adjusted to pH6.0 with triethylamine | |
| Solvent | B: | 50% (v/v) Acetonitrile in Water | |
| Column | : | InertSustain AQ-C18 (3 μm, 1.0 x 50 mm; GL Sciences) | |
| Column | temp. (°C): | RT | |
| Flow rate | (mL/min): | 0.05 | |
| Post-column | Addition: | 2 mM NaOH in 50% (v/v) Acetonitrile | |
| Flow rate | (mL/min): | 0.05 | |
|  |  |  | |
| Gradient |  | 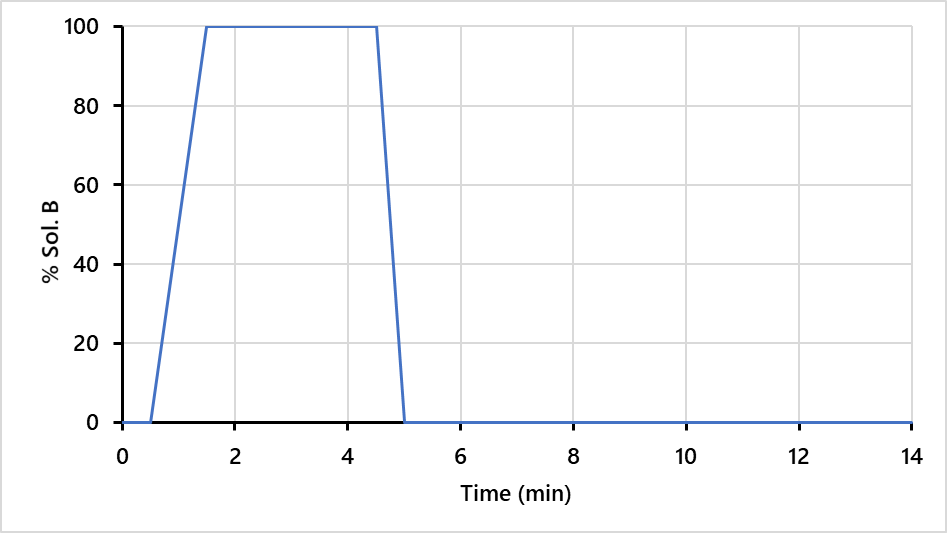 | |
| Time (min) | % sol. B |  |  |
| 0.00 | 0.0 |  |  |
| 0.50 | 0.0 |  |  |
| 1.50 | 100.0 |  |  |
| 4.50 | 100.0 |  |  |
| 5.00 | 0.0 |  |  |
| 14.00 | 0.0 |  |  |
|  |  |  |  |
| (8) Reversed phase HPLC for MS after permethylation | | | |
| Solvent | A: | Water | |
| Solvent | B: | Acetonitrile | |
| Column | : | InertSustain AQ-C18 (3 μm, 1.0 x 10 mm; GL Sciences) | |
| Column | temp. (°C): | 50 | |
| Flow rate | (mL/min): | 0.05/0.1 | |
| Post-column | Addition: | 2 mM NaOH in 50% (v/v) Acetonitrile | |
| Flow rate | (mL/min): | 0.05 | |
|  |  |  | |
| Gradient |  |  | 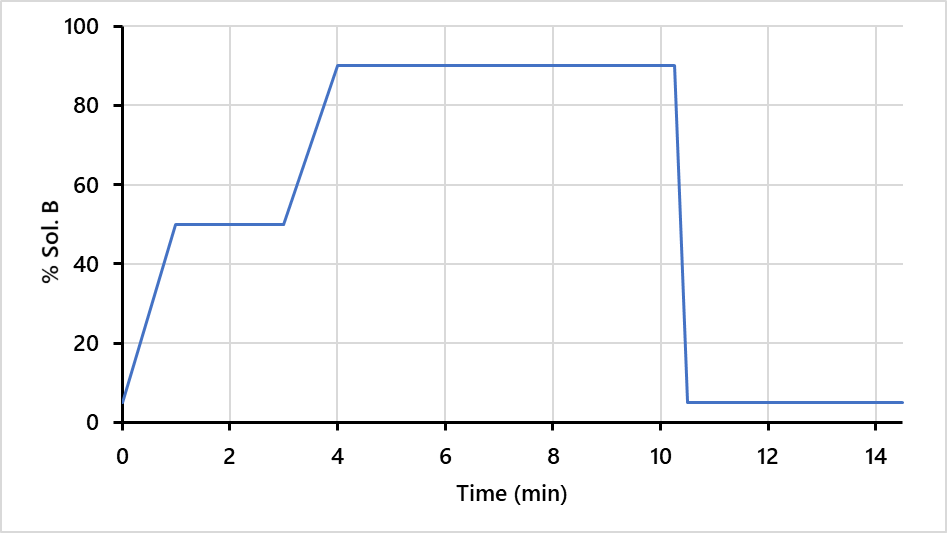 |
| Time (min) | % sol. B | (ml/min) |  |
| 0.00 | 5.0 | 0.1 |  |
| 1.00 | 50.0 | 0.1 |  |
| 2.00 | 50.0 | 0.1 |  |
| 2.25 | 50.0 | 0.05 |  |
| 3.00 | 50.0 | 0.05 |  |
| 4.00 | 90.0 | 0.05 |  |
| 10.00 | 90.0 | 0.05 |  |
| 10.25 | 90.0 | 0.1 |  |
| 10.50 | 5.0 | 0.1 |  |
| 14.50 | 5.0 | 0.1 |  |
|  |  |  |  |
| (9) Reversed phase HPLC for SRM experiment | | | |
| Solvent | A: | 49:1 (v/v), Water / 0.5M acetic acid, adjusted to pH6.0 with triethylamine | |
| Solvent | B: | 24:25:1 (v/v), Water / Acetonitrile / 0.5M acetic acid, adjusted to pH6.0 with triethylamine | |
| Column | : | Shim-pack Scepter C18-120 (3 μm, 2.1 × 150 mm; Shimadzu) | |
| Column | temp. (°C): | 35°C | |
| Flow rate | (mL/min): | 0.25 | |
| Post-column | Addition: | Acetonitrile | |
| Flow rate | (mL/min): | 0.2 | |
|  |  |  | |
| Gradient |  |  | |
| Time (min) | % sol. B | 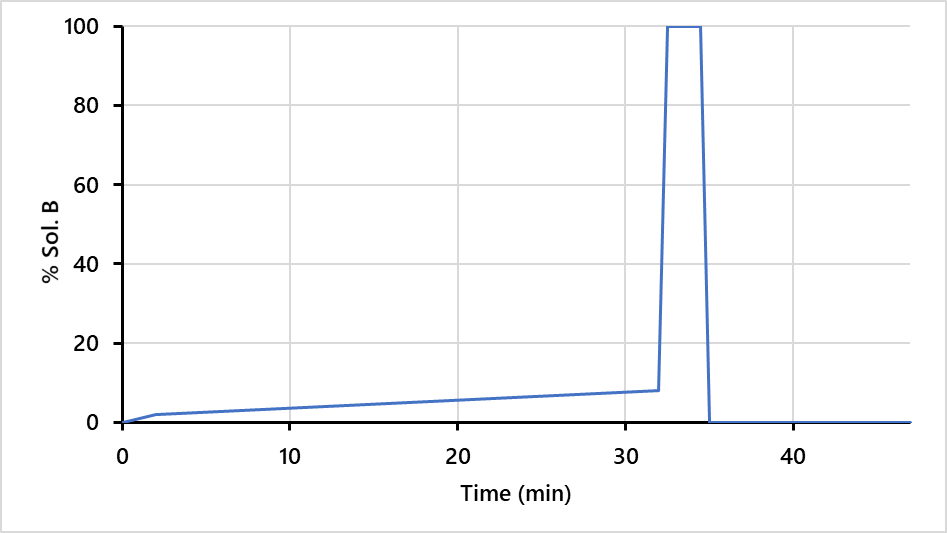 | |
| 0.00 | 0.0 |  |  |
| 2.00 | 2.0 |  |  |
| 32.00 | 8.0 |  |  |
| 32.50 | 100.0 |  |  |
| 34.50 | 100.0 |  |  |
| 35.00 | 0.0 |  |  |
| 47.00 | 0.0 |  |  |
|  |  |  |  |

**Supplementary Table S3.**

The compound-dependent MS parameters for SRM of three PA-glycans

| Glycan structure | Q1 (*m/z*) | Q3 (*m/z)* | CE (V) | DP (V) |
| --- | --- | --- | --- | --- |
| 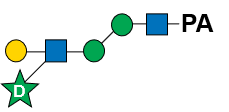 | 1121.4 | **624.3** | 60 | 80 |
|  |  | 989.4 | 50 | 80 |
| 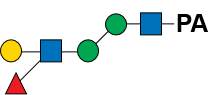 | 1135.5 | **624.3** | 60 | 80 |
|  |  | 989.4 | 50 | 80 |
| 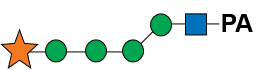 | 1080.4 | **462.2** | 60 | 80 |
|  |  | 300.2 | 60 | 80 |

The quantifier ion of Q3 shown in bold was used for quantification of glycan levels shown in Figure 6. CE and DP denote collision energy and declustering potential, respectively. Entrance potential (EP) and collision cell exit potential (CXP) were set to 10 V and 15 V, respectively.

**
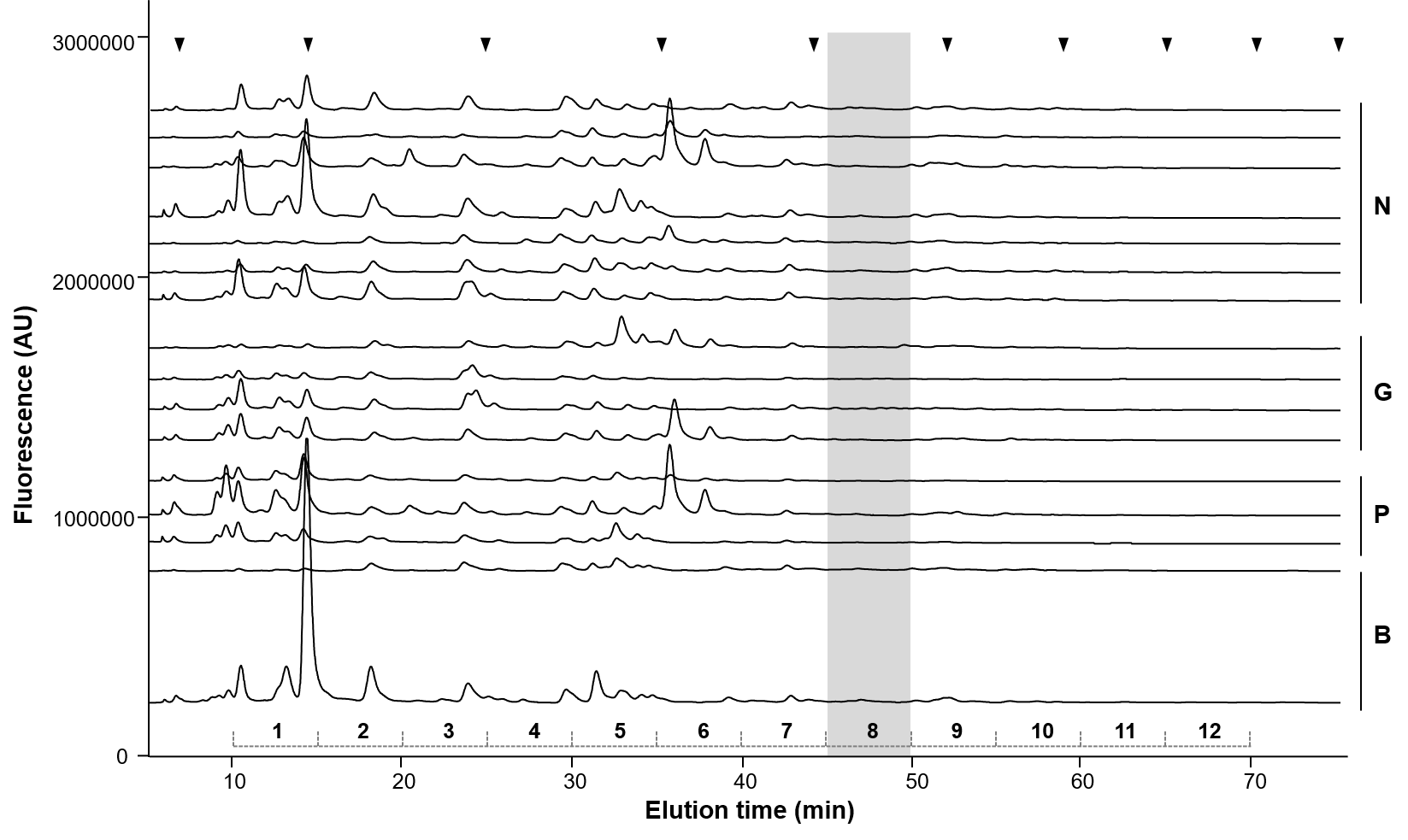
**

**Supplementary Figure S1. Normal phase-HPLC chromatograms of PA-labeled neutral free-glycans prepared from the urine of normal individuals and cancer patients.**

PA-labeled free-glycan mixture in creatinine-adjusted urine from normal individuals and cancer patients were subjected to DEAE anion exchange chromatography. Collected PA-labeled neutral free-glycans were fractionated into 12 (1-12) fractions by NP-HPLC. The RP-HPLC elution pattern of NP-fraction 8, represented by the gray area, is shown in Figure 1. As with the RP-HPLC chromatogram (Figure 1), representative chromatograms of seven normal individuals (N6, N7, N11, N14, N15, N19, N21), four gastric cancer patients (G2, G5, G8, G10), three pancreatic cancer patients (P2, P3, P5) and two bile duct cancer patients (B2, B4) are shown. The black inverted triangles indicate glucose units based on the elution time of PA-labeled isomaltooligosaccharides.


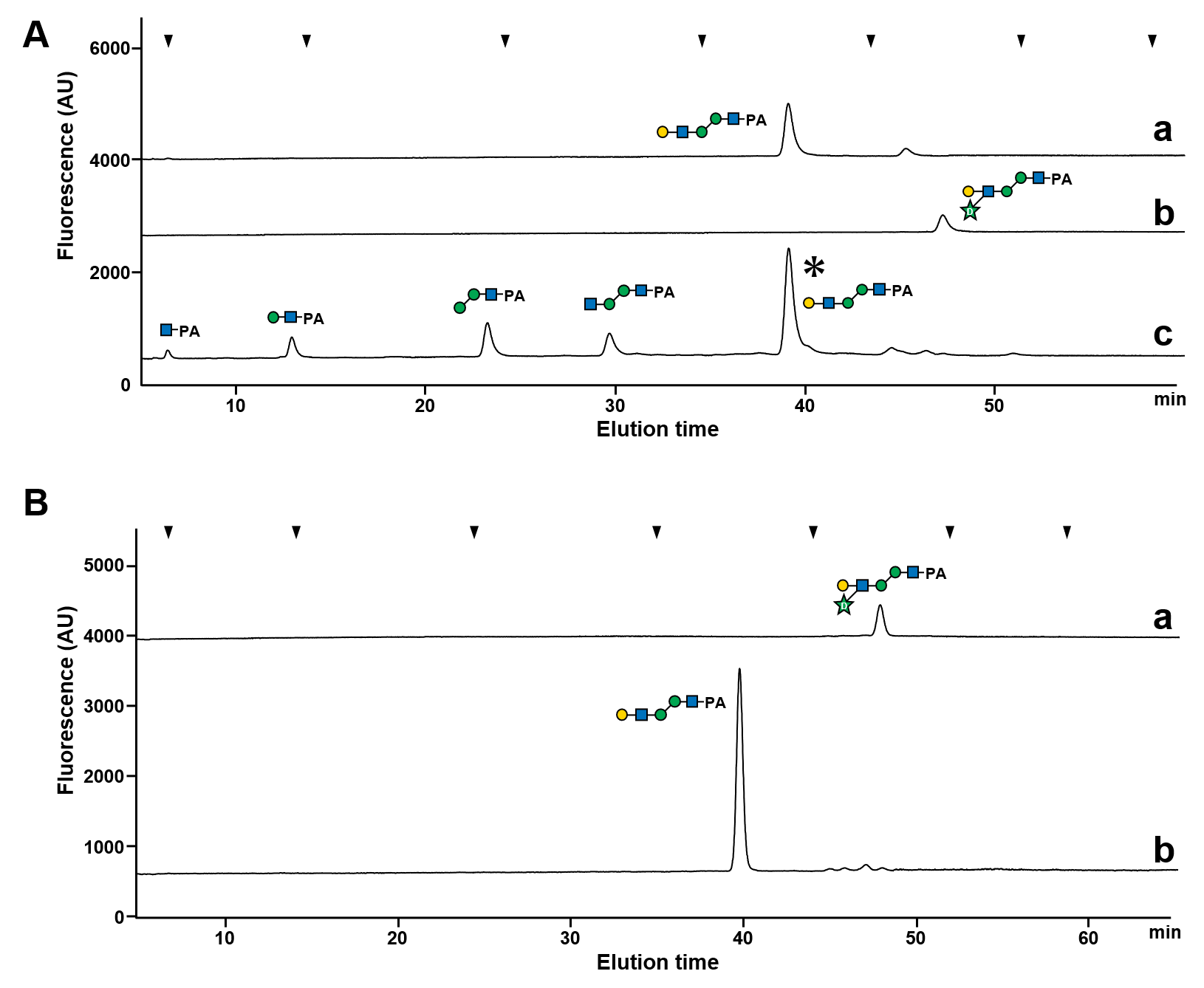


**Supplementary Figure S2. Release of the pentose residue by partial acid hydrolysis or α1,3/4-l-fucosidase digestion.**

The pentose-containing *N*-glycans were subjected to partial acid hydrolysis (A) or α1,3/4-l-fucosidase digestion (B), followed by separation by NP-HPLC. (A) The pentose-containing free-*N*-glycan was subject to partial acid hydrolysis to liberate the pentose monosaccharide. This mild acid hydrolysis reaction liberated almost all the pentose moieties from the original glycan structure. The three chromatograms represent: (a) standard product, Galβ1-4GlcNAcβ1-4Manα1-3Manβ1-4GlcNAc-PA, (b) original *N*-glycan with a pentose residue, (c) hydrolysis products. Backbone structure of this *N*-glycan is indicated by an asterisk in line (c), which was consistent with the standard Galβ1-4GlcNAcβ1-4Manα1-3Manβ1-4GlcNAc-PA shown in line (a).　(B) α1,3/4-l-fucosidase digestion liberated an arabinose residue from the original *N*-glycan structure. (a) and (b) show the chromatograms from the original *N*-glycan with a pentose residue and α1,3/4-l-fucosidase digestion product, respectively. The enzymatic digestion product was consistent with Galβ1-4GlcNAcβ1-4Manα1-3Manβ1-4GlcNAc-PA, as judged by RP-HPLC and MS. The black inverted triangles indicate glucose units based on the elution time of PA-labeled isomaltooligosaccharides.

**
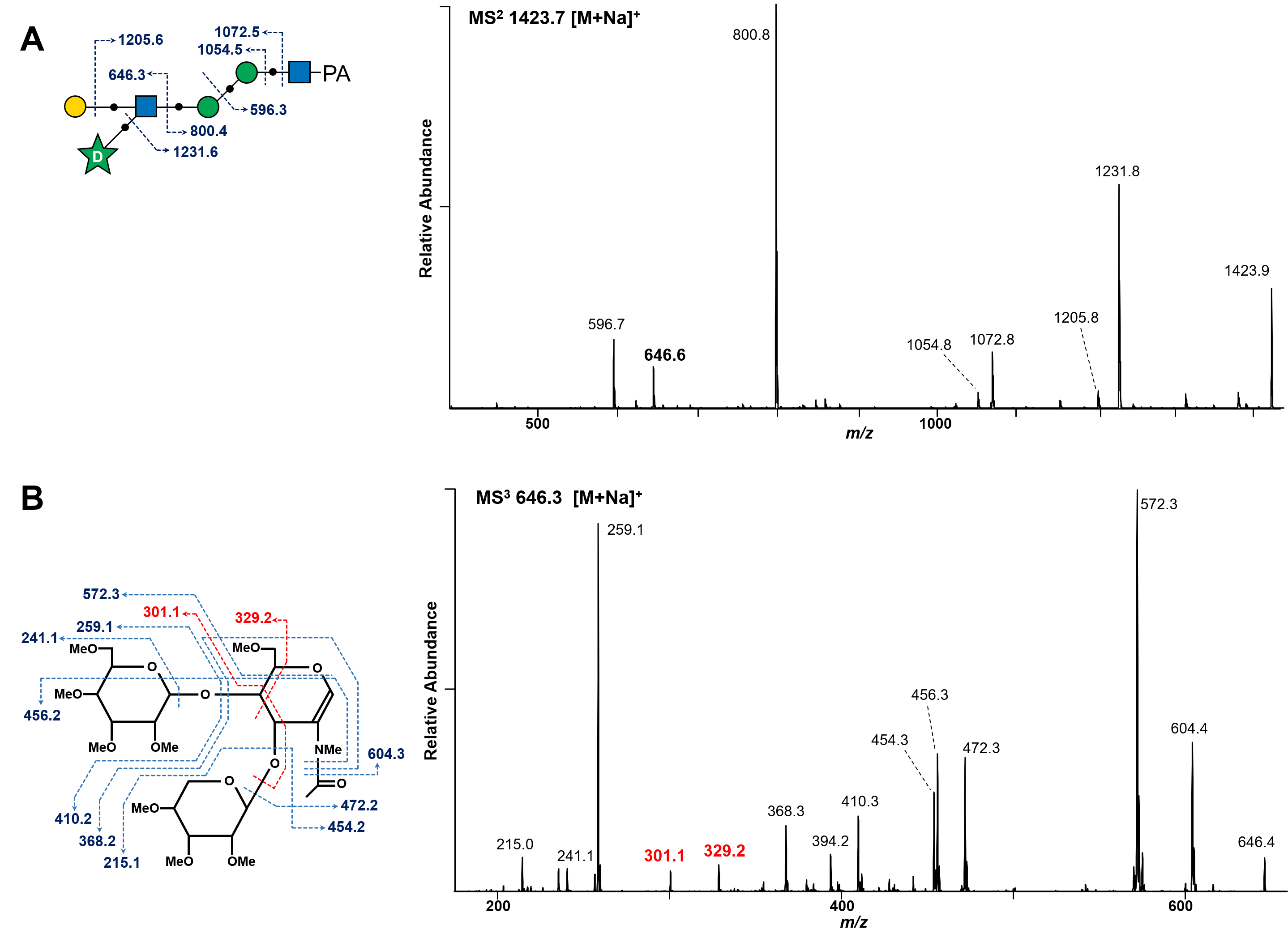
**

**Supplementary Figure S3. LC-MS/MS of the pentose-containing glycan after permethylation.**

(A) MS^2^ spectrum of the permethylated pentose-containing *N*-glycan from a sodiated precursor ion at *m/z* 1423. The MS^2^ fragment ions were assigned as shown in the illustration. Symbol representations of glycans are as follows; galactose, yellow circle; mannose, green circle; GlcNAc, blue square; arabinose, green star. D in the green star indicates the d-enantiomer. (B) MS^3^ spectrum of the antennal B-ion composed of Hex_1_HexNAc_1_Pen_1_ at *m/z* 646 from the MS^2^ spectrum. The 2,4- and 3,5-cross-ring cleavage ions at *m/z* 301 and 329, respectively, which identify the linkages at positions C-3 and C-4, are highlighted in red.


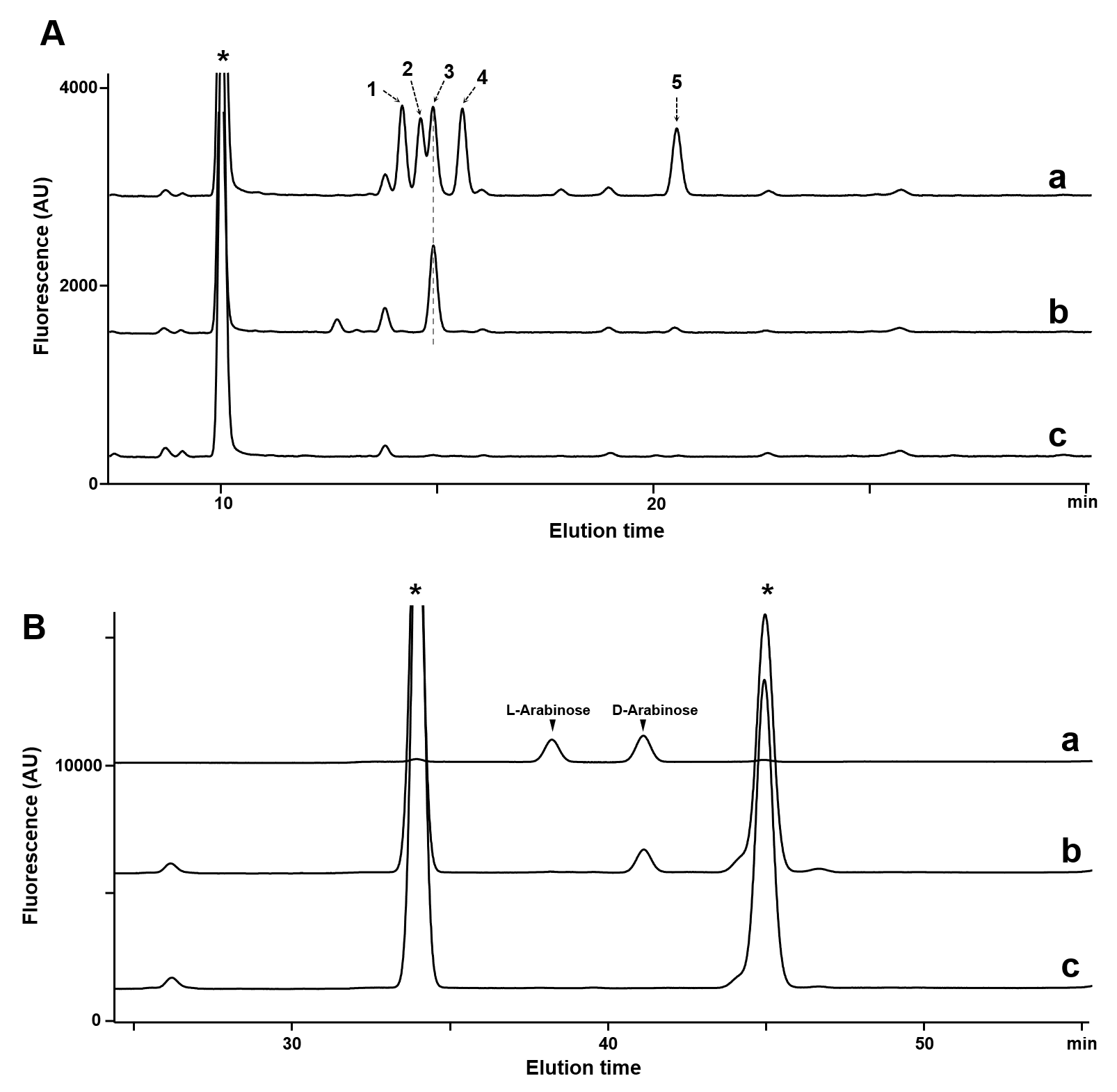


**Supplementary Figure S4. HPLC separation with fluorescent detection was used to identify the pentose monosaccharide released from the pentose-containing glycan.**

HPLC analyses of the derivatized monosaccharides shown in Figure 5 was performed. (A) Chromatogram of 2AB-labeled monosaccharides in RP-HPLC. 2AB-derivated ribose (1), lyxose (2), arabinose (3), xylose (4) and fucose (5) were used as standards (a). Pentose monosaccharide released from the original PA-*N*-glycan analyte by mild acid hydrolysis was labeled with 2AB, and its elution position confirmed by RP-HPLC (b). (c) Chromatogram of the same process without the reaction glycans used as a negative control. Two pmol of the 60 pmol of glycan starting sample for the series of derivatizations was eluted by RP-HPLC. An asterisk indicates unreacted residual free-2AB. (B) Chromatogram of l-TrpNH_2_-labeled monosaccharides in RP-HPLC. A mixture of l-TrpNH_2_-derivatized l-arabinose and d-arabinose was used as standard (a). The arabinose residue released from PA-labeled free-*N*-glycan analyte by partial acid hydrolysis was derivatized with l-TrpNH_2_. Two pmol of the 60 pmol of glycan starting sample for the series of derivatizations was eluted by RP-HPLC (b). (c) Chromatogram of the l-TrpNH_2_-derivatized product without glycans used as a negative control. Asterisks indicate spurious peaks that arose from the reaction process due to the small starting volume of the samples.


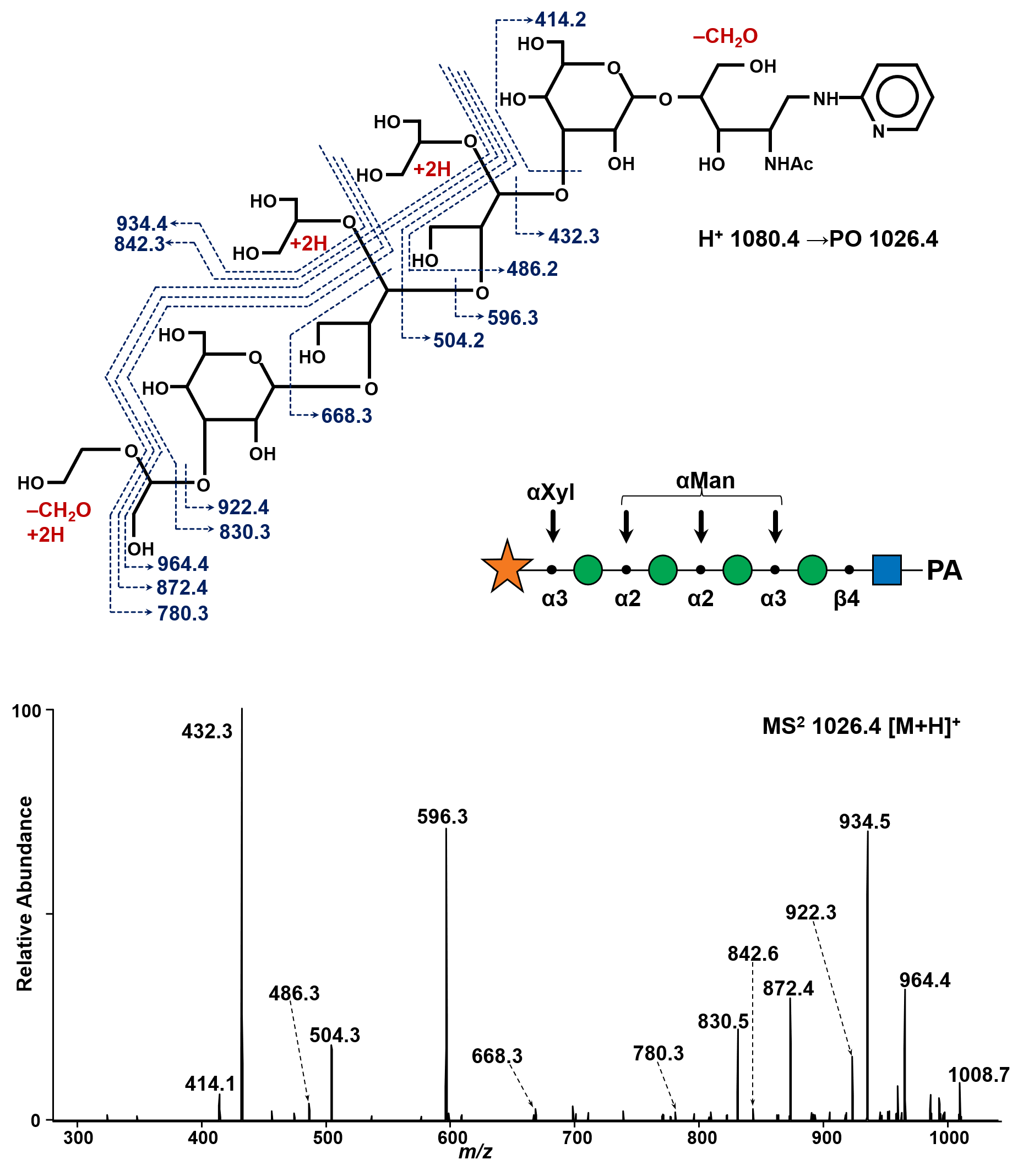


**Supplementary Figure S5. Structural analysis of the xylose-containing glycan.**

The pentose-containing glycan was the major component of the gray dotted peak shown in Figure 1. The MS^2^ spectrum of the periodate-cleaved product at *m/z* 1026 [M+H]^+^ (Hex_4_HexNAc_1_Pen_1_-PA +3×2H, −2×CH_2_O) in positive ion mode MS is shown. The MS^2^ fragment ions were assigned as shown in the illustration. The symbol notation is as follows: mannose for green circles, GlcNAc for blue square, and xylose for orange star. The arrows indicate the position of enzymatic digestion. α-Xylosidase is abbreviated as αXyl, α-mannosidase as αMan.
